# Impact of a tailored exercise regimen on physical capacity and plasma proteome profile in post-COVID-19 condition

**DOI:** 10.3389/fphys.2024.1416639

**Published:** 2024-08-21

**Authors:** Mohammad Mobarak H. Chowdhury, Marie-Noelle Fontaine, Sarah-Eve Lord, Akouavi Julite Irmine Quenum, Marc-André Limoges, Christine Rioux-Perreault, Jean-François Lucier, Dominic O. Cliche, Dominique Levesque, François-Michel Boisvert, André M. Cantin, Hugues Allard-Chamard, Alfredo Menendez, Subburaj Ilangumaran, Alain Piché, Isabelle J. Dionne, Sheela Ramanathan

**Affiliations:** ^1^ Department of Immunology and Cell Biology, Faculty of Medicine and Health Sciences, Université de Sherbrooke, Sherbrooke, QC, Canada; ^2^ Faculty of Physical Activity Sciences, Université de Sherbrooke, Sherbrooke, QC, Canada; ^3^ Research Centre on Aging, Affiliated with CIUSSS de L’Estrie-CHUS, Sherbrooke, QC, Canada; ^4^ Department of Microbiology and Infectious Diseases, Faculty of Medicine and Health Sciences, Université de Sherbrooke, Sherbrooke, QC, Canada; ^5^ Department of Biology, Faculty of Science, Université de Sherbrooke, Sherbrooke, QC, Canada; ^6^ Department of Medicine, Faculty of Medicine and Health Sciences, Université de Sherbrooke, Sherbrooke, QC, Canada

**Keywords:** long COVID, post COVID-19 condition, exercise, physical activity, physical capacity, symptoms, fatigue, oxidative stress

## Abstract

**Background:**

Individuals affected by the post-covid condition (PCC) show an increased fatigue and the so-called post-exertion malaise (PEM) that led health professionals to advise against exercise although accumulating evidence indicates the contrary. The goal of this study is to determine the impact of a closely monitored 8-week mixed exercise program on physical capacity, symptoms, fatigue, systemic oxidative stress and plasma proteomic profiles of PCC cases.

**Methods:**

Twenty-five women and men with PCC were assigned sequentially to exercise (*n* = 15) and non-exercise (*n* = 10) groups. Individuals with no PCC served as a control group. The exercise program included cardiovascular and resistance exercises. Physical capacity, physical activity level and the presence of common PCC symptoms were measured before and after the intervention. Fatigue was measured the day following each exercise session. Plasma and PBMC samples were collected at the beginning and end of the training program. Glutathione and deoxyguanosine levels in PBMC and plasma proteomic profiles were evaluated.

**Results:**

Bicep Curl (+15% vs 4%; *p* = 0.040) and Sit-to-Stand test (STS-30 (+31% vs +11%; *p* = 0.043)) showed improvement in the exercise group when compared to the non-exercise group. An interaction effect was also observed for the level of physical activity (*p* =0.007) with a positive effect of the program on their daily functioning and without any adverse effects on general or post-effort fatigue. After exercise, glutathione levels in PBMCs increased in women but remained unchanged in men. Discernable changes were observed in the plasma proteomics profile with certain proteins involved in inflammatory response decreasing in the exercise group.

**Conclusions:**

Supervised exercise adapted to the level of fatigue and ability is safe and effective in PCC patients in improving their general physical capacity and wellbeing. Systemic molecular markers that accompany physical improvement can be monitored by analyzing plasma proteomics and markers of oxidative stress. Large-scale studies will help identify promising molecular markers to objectively monitor patient improvement.

## 1 Introduction

Even though the majority of COVID-19 infections are relatively mild with recovery typically occurring within 2–3 weeks (Wu and McGoogan, 2020), a significant proportion of COVID-19 patients experience a panoply of long-lasting symptoms after the resolution of the initial SARS-CoV-2 infection. These symptoms, collectively known as the “post COVID-19 condition” (PCC, or “long COVID”), compromise their health-related quality of life and impair daily functioning. PCC is estimated to affect 65 million individuals worldwide including children (Global Burden of Disease Long et al., 2022). The spectrum of PCC manifestations includes, but are not restricted to, cardiac (palpitations, syncope, dysrhythmias and postural symptoms), neuropsychiatric (insomnia, chronic headache, brain fog, defects in memory and mood impairment and pain syndromes) and respiratory (dyspnea and cough), as well as post-exertional malaise (PEM) (Nalbandian et al., 2021). Dyspnea is one of the most common problems associated with PCC (Davis et al., 2021). Up to 25% of PCC patients show a decreased walking distance in the 6-min walking test (6MWT) (Huang et al., 2020; Nalbandian et al., 2021). Chronic fatigue, which is not directly associated with any organ dysfunction, afflicts 78.2% of individuals with PCC at 3 months post-recovery (Kamal et al., 2020). The symptoms may last for months or even years and no curative treatment is yet available for this condition. Patient management is directed towards decreasing the frequency and intensity of clinical manifestations and rehabilitation programs.

Among non-pharmaceutical interventions, tailored exercise therapy is gaining importance to help restore the quality of life in patients living with PCC. In PCC, classical training programs result in PEM with worsening of symptoms (Davis et al., 2021; Jason and Dorri, 2022; Twomey et al., 2022). While the WHO recommendations were not effective in PCC patients, supervised low intensity training or inspiratory muscle training improved the fatigue symptoms, muscle strength and quality of life and had minimal effect on exercise tolerance (Fernandez-Lazaro et al., 2021; Jimeno-Almazan et al., 2023). Moreover, analyses of lived-in experience from patients with PCC suggest that progressive graded physical exercise facilitates recovery (Batatinha et al., 2020; Humphreys et al., 2021), although defined studies incorporating resistance training are not yet reported (Fernandez-Lazaro et al., 2022; Soril et al., 2022). In 2021, Nieman purported that a progressive total workload would be recommended for this population, and that an external surveillance of the patients would be required when returning to physical activity (Nieman, 2021). Also, it is emphasized that intense physical training should be avoided before a full recovery of health to prevent deterioration of general health.

The beneficial effects of exercise on general health, immune responses, metabolic syndrome and central nervous system in infection, old age, neurodegenerative diseases and cancer are well-documented (Agha et al., 2018; Graff et al., 2018; Nieman and Sakaguchi, 2022). With the advent of -omics approaches, comprehensive analyses of exercise-induced changes in physiological and immune parameters are beginning to emerge (Guo et al., 2022). In this proof of principle study, we employed a supervised mixed exercise program on PCC patients for a duration of 8 weeks and analyzed its effects on their physical capacity and evaluated accompanying changes in systemic redox parameters and plasma proteomic profile.

## 2 Materials and methods

### 2.1 Participants recruitment

Participants with PCC were selected from the Biobanque Québécoise de la COVID-19 (Quebec COVID-19 biobank; BQC19). PCC was diagnosed when a patient presents at least one candidate symptom 3 months following a positive COVID-19 PCR test. The candidate symptoms of PCC, as defined by the WHO criteria (WHO, 2021), include fatigue, sleep disturbance, dizziness, chest pain, arrhythmia, dyspnea, cough, abdominal pain, diarrhea, joint pain, myalgia, skin rash, anxiety and depression, headache, loss of taste or smell and brain fog. Individuals presenting a contraindication for exercise, difficulty to perform exercise (walk on a treadmill, stationary exercise, bike cycling or resistance exercise), or with uncontrolled cardiovascular or pulmonary disease were not considered eligible to participate in the study. This study was approved by the ethics review board of the Centre de Recherche du Centre Hospitalier Universitaire de Sherbrooke (protocol # 2022–4415). The study was not a controlled randomized trial (No-PCC group was not included). It was a pilot intervention study.

Eligible participants were contacted by phone and, upon agreement, were invited for a first visit at the research centre. During this visit, informed written consent was obtained, baseline assessments were performed and participants were allocated sequentially to one of the two groups: control (usual care) or exercise (8 weeks of mixed tailored supervised exercise). Height, body weight, waist circumference, physical activity (Physical Activity Scale for the Elderly (Bonetto et al., 2022) questionnaire), and physical capacity were measured before and after the intervention. The Get Active questionnaire (https://csep.ca/2021/01/20/pre-screening-for-physical-activity/) was used to assess participants’ security for physical exercise. This questionnaire considered previous medical history, brief family medical history, as well as the details of the PCC symptoms. The post-intervention visit was set around the same hour of the day and measurements performed in the same order as the first visit. Finally, general fatigue was measured before each exercise session, as a proxy for PEM following the previous session. Otherwise, both groups received usual care, i.e., they continued their regular medical follow-ups at our post-COVID clinic.

### 2.2 Anthropometric measures

Body weight was measured to the nearest 0.02 kg using a SECA 707 electronic scale (Hamburg, Germany). Participants were weighed with light clothes on and shoes off. Body height was measured to the nearest 0.5 cm using a stadiometer (Takei, Tokyo, Japan) with participants standing with their backs against a wall without shoes. BMI was calculated using the formula BMI = weight (kg)/height (m)^2^. Waist circumference was assessed with a flexible tape measure over the iliac crest, measured twice if both measures showed less than 1 cm difference between readings, or measured 3 times. The average of the two closest measures was kept as waist circumference, to the nearest 0.5 cm.

### 2.3 Blood collection and processing

**FIGURE 1:**
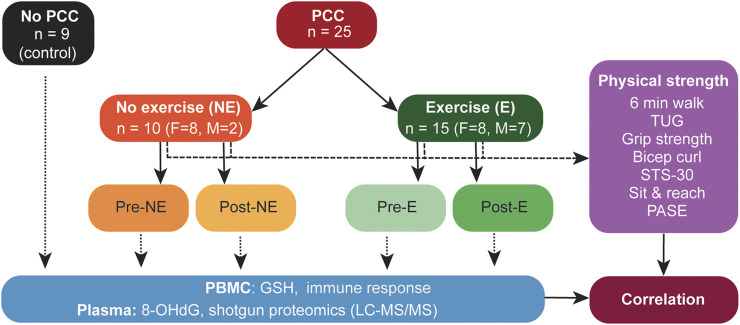
Schematic representation of the study design. PBMC, peripheral blood mononuclear cells; GSH, glutathione; 8-OHdG, 8-Hydroxy-2-Deoxyguanosine; LC-MS/MS, Liquid Chromatography tandem mass spectrometry; STS-30, sit-to-stand test; 6MWT, 6-min walk test; HGS, hand grip strength; TUG, Timed up and go test.

Peripheral blood samples were collected by phlebotomy at recruitment and at post-intervention visit (a week after the last exercise intervention) and were processed within 1 h after collection (Figure 1). Non-fasting blood samples were collected at the same time of the day from antecubital vein using standard vacutainer system. The participants were requested to abstain from alcohol during the 24 h prior to sample collection. Peripheral blood mononuclear cells (PBMC) were isolated over Lymphocyte Separation media (Multicell, CAT# 305–010 CL) density gradient and stored in liquid nitrogen, and plasma fraction stored at −80°C. To measure systemic oxidative stress, freshly isolated 5 × 10^5^ PBMCs were diluted 1:1 with a 10 mM 5,5′dithiobis 2-nitrobenzoic acid (DTNB) solution and kept frozen at −80°C until use.

### 2.4 Exercise program

The exercise group engaged in three non-consecutive mixed exercise sessions per week lasting approximately 50 min, all conducted at the same hour of the day. The 8-week duration program was based on the work of Stavrou et al., and Tanguay et al., (Stavrou et al., 2021; Tanguay et al., 2021), who demonstrated that it was sufficient to observe changes in patients’ health in pulmonary readaptation and telerehabilitation. All exercise sessions were under the supervision of one of the two kinesiologists mandated for this project. We closely monitored fatigue, symptoms and general wellness to document the feasibility of exercising for PCC patients, at a time when health professionals recommended to refrain from physical exertion. In addition, Vital signs (heart rate and blood pressure) were measured before each training session, after the cardiovascular component and at the end of the session, to ensure the participant’s safety throughout the exercise program. Hence, if patients were to report excess fatigue or discomfort, it was intended that the kinesiologist would guide the participant to lower intensity or refrain from specific exercises with the objective of maintaining participation and avoid post-exertion malaise (PEM) or worsening of symptoms.

Each participant started their first training with 5 min of ergocycle and 5 min of treadmill, for a total of 10 min. One minute of aerobic training was added to each session until a maximum of 20 min was reached and maintained until the end (Ahmadi Hekmatikar et al., 2022). To estimate intensity, the participants were instructed to aim for a perceived intensity reaching around 2 to 4 on a 10 points BORG scale and increasing it to a maximum of four points on the Borg scale during the final week (Borg, 1982).

The resistance training (RT) component of the program included exercises recruiting large muscle groups: Squats, lunges, standing hip abduction, bicep curls, shoulder abduction, push-ups, and bridge using free weights and elastic bands. One series of 8–12 repetitions was targeted for each exercise (Ahmadi Hekmatikar et al., 2022). At the first session, a load was selected at which the participants could perform 8–12 repetitions, representing a perceived intensity of 2 to 4 on a 10 points BORG scale and increasing it to a maximum of four points on the Borg scale during the final week. The session ended with 10 min of cool down (very low intensity walk on a treadmill).

Participants had to attend at least 20 of the 24 sessions to be included in final analyses. Adherence was recorded and all participants completed at least 20 sessions. Noteworthy, only one participant presented high levels of fatigue at mid-intervention. It was decided to drop down intensity to the level at the start of the study. With these adjustments, the participant could complete the study and her results were included in the analyses. There were adjustments in the intensity (as measured by the BORG scale for both aerobics and resistance exercise components). The goal was to reach 4 points on a 10-point scale, without going above to avoid triggering post-exertion malaise. However, with training, the intensity (load for resistance training and treadmill speed for aerobics) had to be increased to achieve four points on BORG. Hence, when participants fell below 4 at the end of a given exercise, intensity would be slightly increased on the next training session. As we didn’t do a maximal strength test with our participants, we mostly used stretch bands (horizontal pull, hip abduction, shoulder abduction) and body weight as resistance (squats, push ups, plantar flexion, lunges). Hence, when participants fell below 4 at the end of a given exercise, intensity would be slightly increased on the next training session by increasing the resistance and/or the number of repetitions. Free weights were used for bicep curls and, for a few participants only, we added free weights at some points to hold during the squat exercise. Elastic bands were used for standing hip abduction and shoulder abduction.

#### 2.4.1 Physical capacity

Physical capacity was assessed by six different tests from the senior fitness test battery (6-min walk test-6MWT, TUG, Grip strength, arm strength, 30 sec-sit to stand-STS-30, sit and reach) (Rikli and Jones, 2013). These tests were selected as they are accessible for most people, even for the participants strongly affected by PCC. As these tests are applicable to the elderly population, we hypothesized that the same battery of tests can be applied to PCC patients given their reduced exercise tolerance.

##### 2.4.1.1 Timed up and go (TUG) test

A chair was placed 3 m away from a mark on the floor. Participants were seated on a straight-back chair without armrests and were instructed to rise and walk up to and around the mark to come back and sit on the same chair. The test started when the participants lifted from the sitting position and ended when the participants were back on the chair. Participants repeated the test once, and the fastest time in seconds was kept as the value.

##### 2.4.1.2 6-Min walk test (6MWT)

Participants were asked to walk as fast as possible around two cones placed 100 feet apart for the duration of 6 min, without talking and stopping only if needed. Heart rate was monitored (Polar H10, Canada), and the total travelled distance in meters was calculated at the end of the test.

##### 2.4.1.3 Hand grip strength (HGS) test

Participants were standing up, arms to their sides, and asked to press as hard as they could on the handle while breathing out the air in their lungs. The measure was performed standing, with the arm in full extension, hand directed to the floor. All tests were performed in the same position. A 60 sec rest time was allowed between measurements. Testing was done twice on each side, by alternating sides using a dynamometer (±0.5 kg, hydraulic grip dynamometer, Lafayette hand dynamometer; Model 78,010). The average strength in kg was calculated for the four measures.

##### 2.4.1.4 Bicep curl test

Upper body performance was assessed by the Bicep Curl Test as a surrogate of muscle endurance. Participants were sitting on a chair without armrests and asked to complete a maximum of repetitions of a bicep curl for 30 sec, using 2.27 kg for women and 3.63 kg for men. Repetitions would only count if the elbow was completely extended and bent. Only the dominant side was tested, and the total number of completed repetitions was noted.

##### 2.4.1.5 30 sec sit-to-stand (STS-30) test

Lower limb capacity was assessed using the 30-sec sit-to-stand (STS-30). The test was performed on a standardized chair from a seated starting position, arms crossed on the chest. The test started when the participant initiated the first standing motion. The test ended after 30 sec, with the total number of completed repetitions (return to start position) as the result.

##### 2.4.1.6 Sit and reach test

Lower body flexibility was assessed with the Sit & Reach Test. Participants sat on a chair and were asked to extend one leg forward, while keeping the other grounded. Hands were disposed one on top of the other. When breathing out, they would move their hands as close to their foot as possible while looking forward. A measure in cm would then be taken with a ruler between the tip of third finger and the tip of the shoe. Both sides were measured and averaged as a result.

##### 2.4.1.7 Physical activity level

Physical activity level (PASE questionnaire (Washburn et al., 1993)) was measured before and after the intervention. This tool was selected because of its validity in individuals with somewhat limited capacity, i.e., older adults. At the time of the study, very little was known about the physical condition of long COVID individuals. Leisure time, household, and work-related activities during the past week are reported by the participant, excluding the exercise program done if they were in the exercise group. First, daily activities were scored in compliance with the intensity and time of reported activities. The sum of each question is a global score representing physical activity level. Noteworthy, the measure taken after the intervention did not include the exercise sessions performed during the program.

##### 2.4.1.8 Acute fatigue and symptoms

Participants of the exercise group were questioned on their fatigue level before each training session and asked to rate it on a scale of 1 to 5 (1 = no fatigue, 5 = extreme fatigue). This assessment served to adjust the training intensity. Average values were calculated for each week.

Participants were also questioned regarding the perceived presence (yes or no) of 8 common PCC symptoms: altered taste and smell, anxiety, neurological symptoms, general pain, asthma, resting shortness of breath and shortness of breath on exertion. The number of symptoms (number of yeses) was summed for baseline and post-intervention.

### 2.5 Immune responses to SARS-CoV-2 spike protein

Cryopreserved PBMCs were thawed, and an activation-induced marker (AIM) assay was carried out to detect T cell responses to spike protein as described (Limoges et al., 2023a). In brief, cells were plated in quintuplicates and incubated overnight at 37°C in CO_2_ incubator. PepTivator^®^ SARS-CoV-2 Prot_S peptide pool (Miltenyi Biotec) was added at a concentration of 1 μg/mL according to product specification. Cells were collected 18 h later and processed for flow cytometry. Spike peptide pool-specific responses were calculated by subtracting the values obtained in cultures without the added peptides from the same experiment. RBD-specific B cells were identified by labelling freshly thawed PBMCs with anti-RBD probes as described (Limoges et al., 2023a; Limoges et al., 2023b) in the presence of a panel of markers to characterize the different B cell subsets (Supplementary Table S1).

### 2.6 Plasma proteomics

The plasma samples were processed for proteomic analyses as described (Frion et al., 2023). In brief, 75 μg of plasma proteins were reduced in 50 μL of 10 mM HEPES-KOH pH 7.5, 8 M urea by adding dithiothreitol to a final concentration of 5 mM and by heating at 95°C for 2 min, followed by a 30 min incubation at room temperature. Alkylation of proteins was then carried out by adding chloroacetamide (Sigma-Aldrich, Saint-Louis) to a final concentration of 7.5 mM and 20 min incubation at room temperature away from light. Urea was diluted to a final concentration of 2 M by adding 50 mM NH_4_HCO_3_ (Sigma-Aldrich, Saint-Louis). Proteins were digested by adding 1 μg of Pierce mass spectrometry (MS)-grade trypsin (Thermo Fisher Scientific, Waltham) and incubated overnight at 30°C while shaking. Peptides were purified with micropipette tips containing a C18 column (EMD Millipore, Burlington, VT), concentrated by centrifugal evaporation and then resuspended in 1% formic acid. Peptides were quantified using a NanoDrop spectrophotometer (Thermo Fisher Scientific, Waltham, MA).

For mass data acquisition in data independent acquisition (DIA) mode, 250 ng of peptides from each sample were injected into an HPLC (nanoElute, Bruker Daltonics) and loaded onto a trap column with a constant flow of 4 μL/min (Acclaim PepMap100 C18 column, 0.3 mm id × 5 mm, Dionex Corporation), and then eluted onto an analytical C18 Column (1.9 µm beads size, 75 μm × 25 cm, PepSep) heated at 50°C. Peptides were eluted for 2 h over gradient of acetonitrile (5%–37%) in 0.1% FA at 400 nL/min while being injected into a TimsTOF Pro ion mobility mass spectrometer equipped with a Captive Spray nano electrospray source (Bruker Daltonics). Data was acquired using diaPASEF mode (Meier et al., 2020). Briefly, for each single Trapped Ion Mobility Spectrometry (TIMS; 100 ms) in diaPASEF mode, 1 mobility window consisting of 27 mass steps (m/z between 114 and 1,414 with a mass width of 50 Da) per cycle (1.27 sec duty cycle) was used. These steps cover the diagonal scan line for +2 and +3 charged peptides in the m/z-ion mobility plane.

### 2.7 DIA-NN analysis

The MS data were processed with DIA-NN (Demichev et al.,, 2020), an open-source software suite for DIA/SWATH data processing (https://github.com/vdemichev/DiaNN, version 1.8.1) installed in an Apptainer container (https://apptainer.org/) using docker image provided on docker hub https://hub.docker.com/layers/biocontainers/diann/v1.8.1_cv1/images). Analysis was performed using default parameters in addition to permissive cleavages (up to two max) and protein N-terminal methionine as variable for the *in silico* digestion.

The human FASTA proteome UP000005640 was downloaded from the Uniprot website (https://ftp.uniprot.org/pub/databases/uniprot/current_release/knowledgebase/reference_proteomes/Eukaryota/UP000005640/). The reference proteome contained a total of 96,418 proteins. For the FASTA search, DIA-NN was instructed to perform an *in silico* digest of the sequence database. A mass tolerance accuracy of MS1 and MS2 of 20 ppm was used for precursor and fragment ions, respectively. Minimum and maximum were set for peptide length (7–30 amino acids), and precursor charge (1–5), precursor m/z (100–1,700) and fragmentation m/z (100–1,500) for *in silico* library generation or library-free search. For the reanalysis, MBR (match between run) and smart profiling were enabled to create a spectral library from the DIA data. Carboxyamidomethylation (unimod4), and oxidation (M) (unimod35) were set as fixed modifications and N-terminal protein acetylation was set as a variable modification.

All post-analyses were performed using R v4.3.2 (R core team, www.R-project.org) to generate the DIA-NN results output file (unique_genes_matrix.tsv). This matrix represents genes identified and quantified using only proteotypic (gene-specific), peptides. They are filtered at 1% FDR, using global *q*-values for protein groups and both global and run-specific *q*-values for precursors. All heatmaps where produced with clustering method set to complete and distance method to Euclidean. PCA were plotted using the ggbiplot (https://github.com/vqv/ggbiplot) library.

### 2.8 Total glutathione measurement in PBMC

Quantitative determination of total glutathione levels (GSH + GSSG) in PBMC was performed using an enzymatic assay (Akerboom and Sies, 1981). Samples were sonicated for 3–5 sec on ice and passed through a 10K-centrifugal filter unit (Amicon). The undiluted filtrate was added to the enzymatic reaction mix composed of 100 mM potassium phosphate, 5 mM EDTA, 25 mU glutathione reductase and 250 μM NADPH. The formation of the TNB chromophore was recorded on a spectrophotometer at 412 nm for 2 min. A curve generated from standards of known concentrations of glutathione disulfide (GSSG 0.5–8 μM) was used to determine glutathione concentrations in test samples.

### 2.9 ELISA assays

The concentrations of free 8-hydroxy-2' -deoxyguanosine (8-OHdG) in plasma was measured using competitive assay (Stressmarq Biosciences, Canada; Cat# SKC-120A) following the manufacturer’s protocol. The 8-OHdG levels were established utilizing the standard curve and expressed in ng/mL. Cystatin 3 (CST3) (Sinobiologicals, China; Cat# SEK10439) and S100A8 (Proteintech, United States; Cat# KE00177) was determined in the plasma using the kits according to the manufacturers’ instructions.

### 2.10 Statistical analyses

Results are presented as mean ± standard deviation (SD). Baseline similarities between groups were assured using an independent *t*-test. Repeated measures ANOVA were used to determine the effect of the intervention on clinical outcomes where an interaction effect indicates a significantly greater change in one group compared to the other. The number of symptoms were compared pre and post intervention in each group using a McNeymar analysis (SPSS 20.0 for Windows, Chicago, IL, United States, statistical significance was set at *p* ≤ 0.05). GraphPad Prism version 9.5 software was used for statistical analyses of systemic oxidative stress and plasma proteomic profiles (Statistical comparisons were carried out with the Wilcoxon test) and to generate graphics.

## 3 Results

### 3.1 Baseline physical, anthropometric and clinical parameters of the study population

Out of 340 PCC patients contacted, 32 individuals fulfilled the eligibility criteria and agreed to participate in this study. One participant dropped out in the exercise group because the research centre was too far. Six were excluded for diverse reasons including COVID-19 re-infection, failure to meet the inclusion criteria at visit 1, failure to show up at first visit, hip injury, cardiac complications and cancer diagnosis. The baseline characteristics of the 25 PCC participants who completed the study (controls n = 10 and exercise n = 15, mean age 51,5 ± 13,8 years) are shown in Table 1, and the overall study design is outlined in Figure 1. The exercise and the no-exercise groups were similar for all tested physical parameters at baseline, except for weight and body mass index (BMI; Table 2). No adverse events occurred during testing or training sessions. While body weight and waist circumference were not significantly different between groups, BMI tended to significantly improve in the exercise group only. Resting heart rate decreased significantly in both groups to a comparable extent (Table 2). The nine no-PCC controls were volunteers from hospital workers.

**TABLE 1:** Baseline clinical characteristics of study participants.

Variables	No-PCC n = 9	PCC^a^ n = 25
Male Sex n (%)	3 (33.3)	9 (36.0)
BMI (kg/m^3^) mean (SD)	ND	28.3 (4.3)
Mean age years (SD)	46.4 (9.3)	51.5 (13.8)
Comorbidities n (%)	ND	
Obesity		9 (15.6)
Hypertension		10 (15.6)
Diabetes		2 (6.3)
dyslipidemia		7 (9.0)
Autoimmune diseases		3 (9.3)
number of PCC symptoms median value (range)	N/A	4 (2–9)
Post-COVID-19 symptoms duration mean (months, range)	N/A	17 (1–23)
Number of fully vaccinated patients pre-infection	N/A	10/25

^a^ According to WHO classification (WHO, 2021).

No-PCC, convalescent individuals without PCC; PCC, convalescent individuals with PCC; PCC, post-covid condition; SD, standard deviation; ND, not documented- They were healthy research co-workers with no known co-morbidities; N/A, not applicable.

**TABLE 2:** Baseline physical, anthropometric and clinical parameters of the PCC patients in the no-exercise and exercise group before and after the exercise intervention.

	Exercise (n = 15)		No-exercise (n = 10)		Time effect *p*-value	TimeXgroup effect *p*-Value
	7 men, 8 women		2 men, 8 women			
	Pre	Post	Pre	Post		
Age (yrs)	51.3 ± 12.2	—	51.8 ± 16.7	—		
Duration of PCC in months median value (range)^a^	20 (1–22)	—	10.5 (3–23)	—		
Vaccinated before infection	6/15	—	5/10	—		
Vaccinated before recruitment (1–4 doses)	15/15	—	10/10	—		
Weight (kg)*	81.2 ± 10.2	80.85 ± 9.9	72.8 ± 9.3	73.3 ± 10.0	*n.s*	*n.s*
Height (cm)*	167.3 ± 7.3	—	164.1 ± 6.1	—		
BMI (kg/m^2^)*	29.1 ± 4.3	28.96 ± 4.1	27.2 ± 4.3	27.65 ± 4.5	*n.s*	*p = 0.077*
Waist circumference (cm)*	101.7 ± 8.7	101.11 ± 7.6	92.8 ± 11.5	92.5 ± 11.82	*n.s*	*n.s*
Systolic Blood pressure (mmHg)*	122.7 ± 12.6	122.73 ± 11.4	119.6 ± 11.9	118.0 ± 12.2	n.s	n.s
Diastolic blood pressure (mmHg)*	77.3 ± 7.6	79.13 ± 6.4	78.2 ± 7.6	76.30 ± 6.8	n.s	n.s
Resting heart rate (BPM)*	77.1 ± 10.3	74.73 ± 10.8	82.1 ± 9.8	75.80 ± 1.0	*p = 0.057*	*n.s*

*Mean ± SD. *p* values in Italic indicate tendencies. n.s, not significant; BPM, beats per minute.

^a^ Baseline was calculated from 3 months post-infection.

### 3.2 Positive impact of tailored exercise regimen on physical capacity and overall physical activity

**FIGURE 2:**
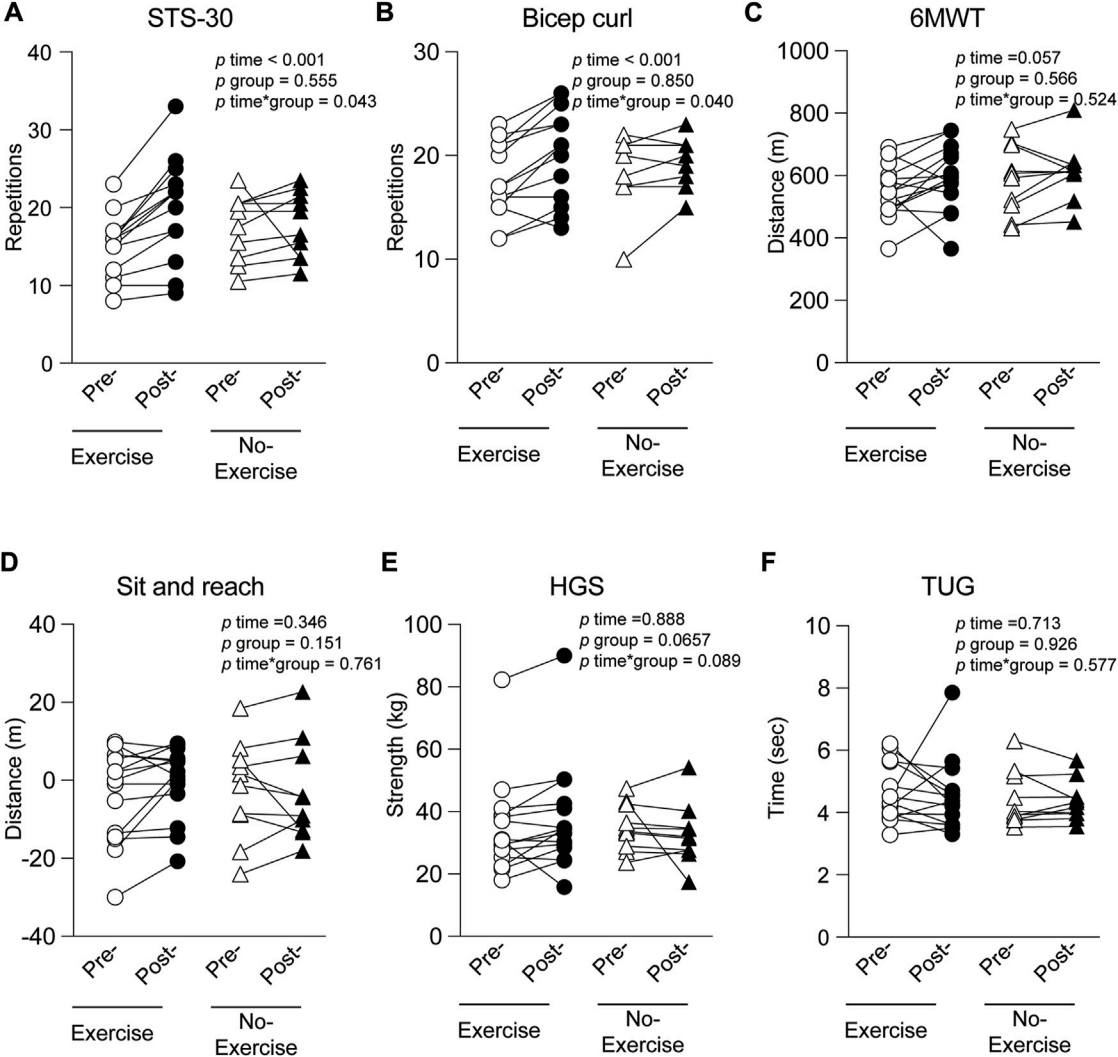
Evaluation of different exercise associated parameters at inclusion (pre-) and at the end of the study (post-) in the group that had physical training intervention (Exercise) or not (No-exercise). **(A)** STS-30-sit-to-stand test; **(B)** bicep curl; **(C)** 6MWT-6-min walk test; **(D)** Sit and reach; **(E)** HGS-hand grip strength; **(F)** Tug-Timed up and go test.

After exercise intervention, the sit-to stand test (+31% vs. +11%; *p* = 0.043) (Figure 2A) and bicep curl test (+15% vs. 4%; *p* = 0.040) (Figure 2B) significantly improved with time but to a greater extent in the exercise group as compared to the control group (all *p* < 0.001). The exercise group showed a closely significant improvement over time for 6-MWT (*p* = 0.057) (Figure 2C) and a tendency for a significantly greater progress for HGS (+8% vs. −7%; *p* = 0.089) (Figure 2E). A significant correlation was observed between the improvement for the bicep curl test and the walking distance of the 6-MWT (r = −0.467, *p* = 0.022) or the STS-30 test (r = 0.535, *p* = 0.006). Statistical power was not sufficient to determine any sex effect on the exercise intervention. A significant interaction effect was also observed for the level of physical activity (*p* = 0.007) as the Physical Activity Scale for the Elderly (PASE) questionnaire score increased for the exercise group (pre: 181.90 ± 82.53; post: 234.47 ± 90.47), compared to a decrease in the control group (pre: 229.20 ± 91.82 and post: 202.80 ± 87.05), supporting an effect of the exercise intervention on physical activity outside of the training program.

### 3.3 Minimal impact of exercise regimen on PCC symptoms and fatigue

**FIGURE 3:**
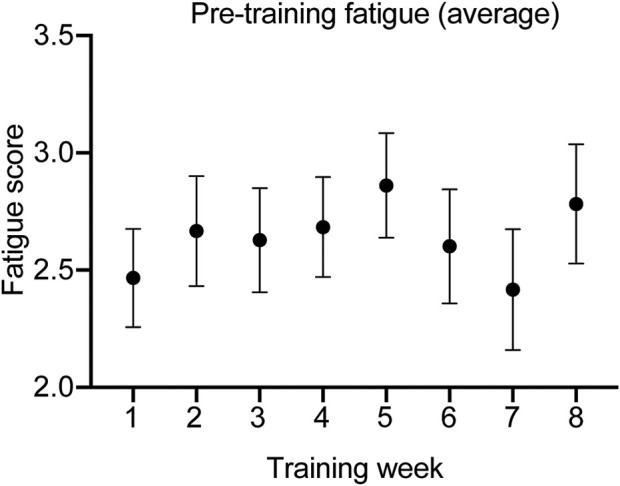
Fatigue score reported over time.

The exercise regimen had no significant impact on altered taste and smell, anxiety, neurological symptoms, general pain, asthma, resting shortness of breath and shortness of breath on exertion. The number of symptoms before and after the intervention was also not significantly different between groups. General fatigue was measured in the exercise group. The averaged fatigue score (out of 5) of the three training days is displayed week by week in Figure 3. Statistical comparison between weeks 1 and 8 of the training program revealed that the tailored intervention had no significant impact on general fatigue. Although PEM was not measured, an acute elevated fatigue would have been a sign of potential PEM, which we did not observe. Hence, the exercise program can be considered promising since PEM has been observed following classical training programs in the PCC group (Wright et al., 2022; Norweg et al., 2023).

### 3.4 Exercise regimen does not adversely affect spike protein specific T and B cells

**FIGURE 4:**
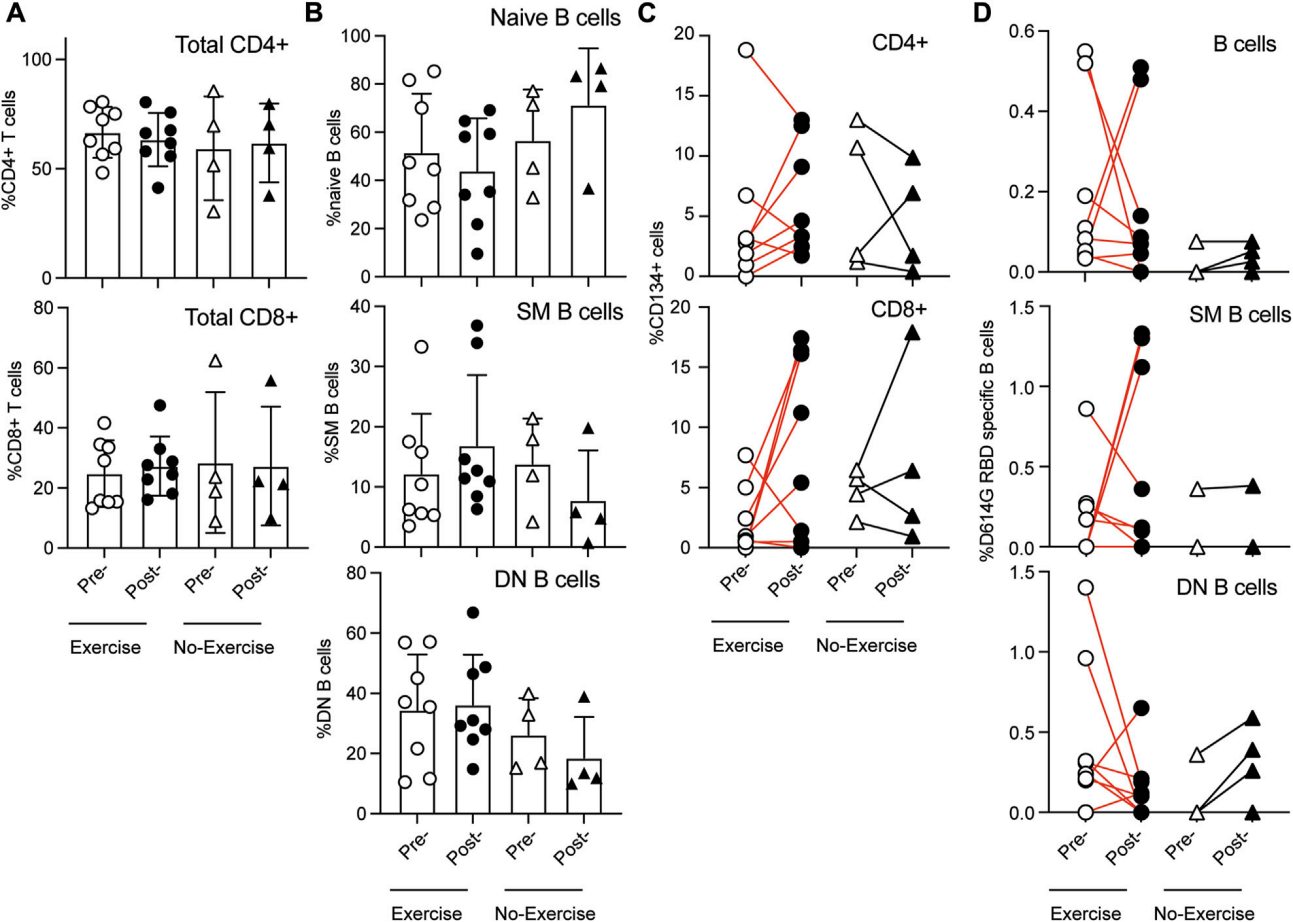
Proportion of T and B cell subsets in the peripheral blood samples at inclusion (pre-) and at the end of the study (post-) in the group that had physical training intervention (Exercise) or not (No-exercise). **(A)** Proportion of CD4^+^ and CD8^+^ cell subsets in T lymphocytes; **(B)** Proportion of naïve (IgD+), switched memory (SM, IgD-CD27^+^) and double negative (DN, IgD-CD27^−^) B cells subsets within CD19^+^ B cells. T and B cell response to spike protein at inclusion (pre-) and at the end of the study (post-) in the group that had physical training intervention (Exercise) or not (No-exercise). **(C)** PBMC were stimulated with pooled spike peptides and specific response was determined (AIM assay) in CD4^+^ and CD8^+^ T cell subsets. CD134+ (OX40) was used to identify spike-specific T cell responses. **(D)** PBMCs were labeled with D614G-RBD to label RBD-specific B cells in total, switched memory (SM) and double negative (DN) subsets. Statistical comparisons were carried out with the Wilcoxon test.

As physical training has been shown to influence immune responses, we analyzed the T and B cell responses to spike peptides in a subgroup of PCC patients (Exercise-8; No-exercise-4). All of them had received two or three doses of vaccines and the last vaccine dose was 3–5 months before recruitment. None of them had received any vaccine during the duration of the study. The gating strategy used for identifying T and B cell subsets is shown in Supplementary Figure S1. The frequency of major T and B cell subsets were comparable between the two groups at both the time points (Figures 4A, B).

PBMCs were stimulated for 24 h with a SARS-CoV-2 Prot_S peptide pool and analyzed using an activation-induced marker assay (AIM assay) by assessing CD134 (OX40) positivity in combination with either CD69 or CD25 (Reiss et al., 2017) (Supplementary Figure S1A). More than half of the PCC patients gained T cell reactivity after 2 months of exercise training (5 out of 8), whereas most cases in the no-exercise group showed a decrease in both the CD4^+^ and CD8^+^ T cell subsets, although the number of cases is too few for a quantitative comparison (Figure 4C). These responses observed in the PCC exercise group are comparable to what was observed in vaccinated healthy individuals as reported by us (Limoges et al., 2023a). As different parameters have been reported in the literature to assess T cell reactivity, it was not possible to compare with the results reported in the literature (Sette et al., 2023). Previously we reported that the B cell responses to SARS-CoV-2 spike RBD region was comparable between the infected individuals with and without PCC (Limoges et al., 2023b). Here we assessed whether the tailored exercise intervention altered the frequency of circulating B cells specific to D614G RBD, a B cell epitope present in the initial COVID-19 vaccines. D614G RBD-specific B cells were identified using labeled, first-generation vaccine strain SARS-CoV-2 RBD peptide (D614G-RBD, Figure 4D). D614G RBD specific total and switched memory (SM, IgD-CD27^+^; memory B cells generated in germinal centers in secondary lymphoid organs) B cell frequency generally decreased in the exercise group with a few cases showing an increase, whereas these B cells remained fewer in number and unchanged in the no-exercise group (Figure 4D). Notably, the double negative (DN, IgD-CD27^−^; memory B cells generated outside germinal centers) B cells showed a tendency to decrease in the exercise group but to increase in the no exercise group. Even though these differences suggest a shift in RBD-specific B cell responses in the exercise group, the low sample numbers preclude any meaningful statistical comparison between the exercise intervention and control groups. Nonetheless, these observations indicate that the exercise regimen is unlikely to adversely affect the overall immune responses to the SARS-CoV-2 antigens.

### 3.5 Exercise-induced oxidative stress response does not adversely affect the redox homeostasis

**FIGURE 5:**
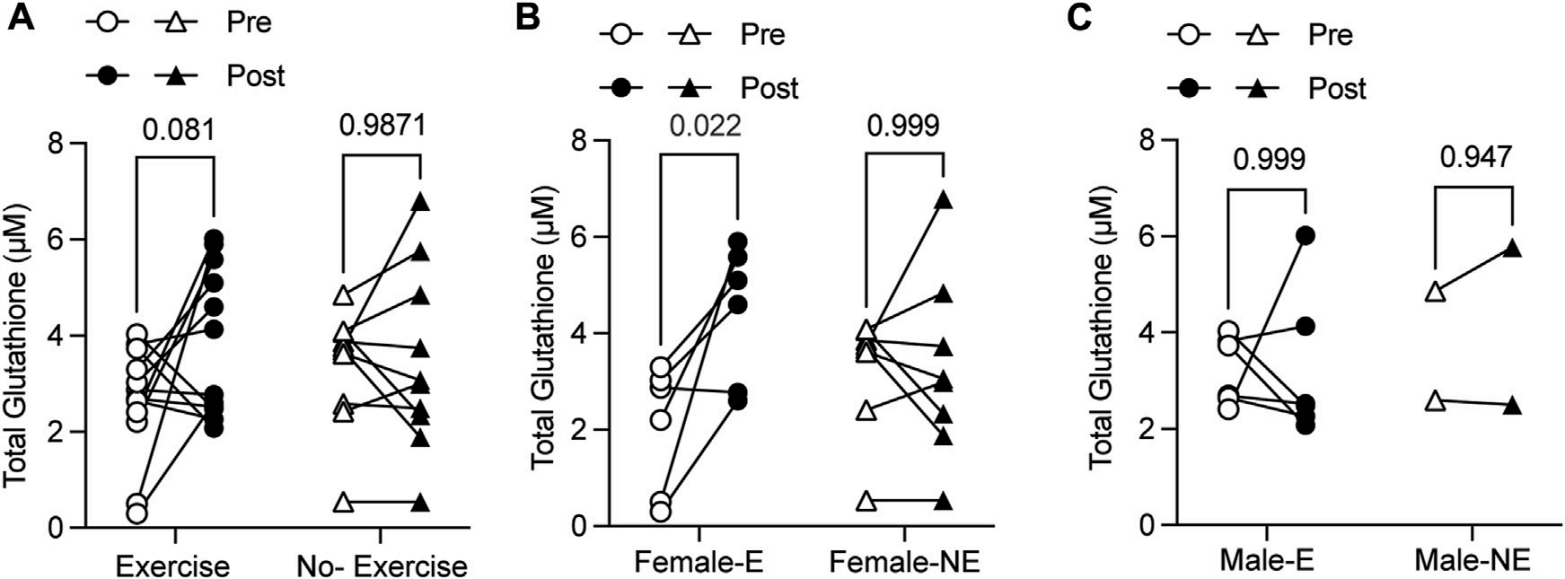
Total GSH levels in PBMCs isolated from exercise (E) and no-exercise (NE) PCC groups measured by enzymatic assay. **(A)** Paired analysis of pre- and post- GSH levels within exercise and no-exercise PCC groups. **(B, C)** Paired analysis of GSH levels among female **(B)** and male **(C)** PCC subjects within exercise (-E) and no-exercise (-NE) groups. Statistical comparisons were carried out with the Wilcoxon test. GSH, glutathione.

**FIGURE 6:**
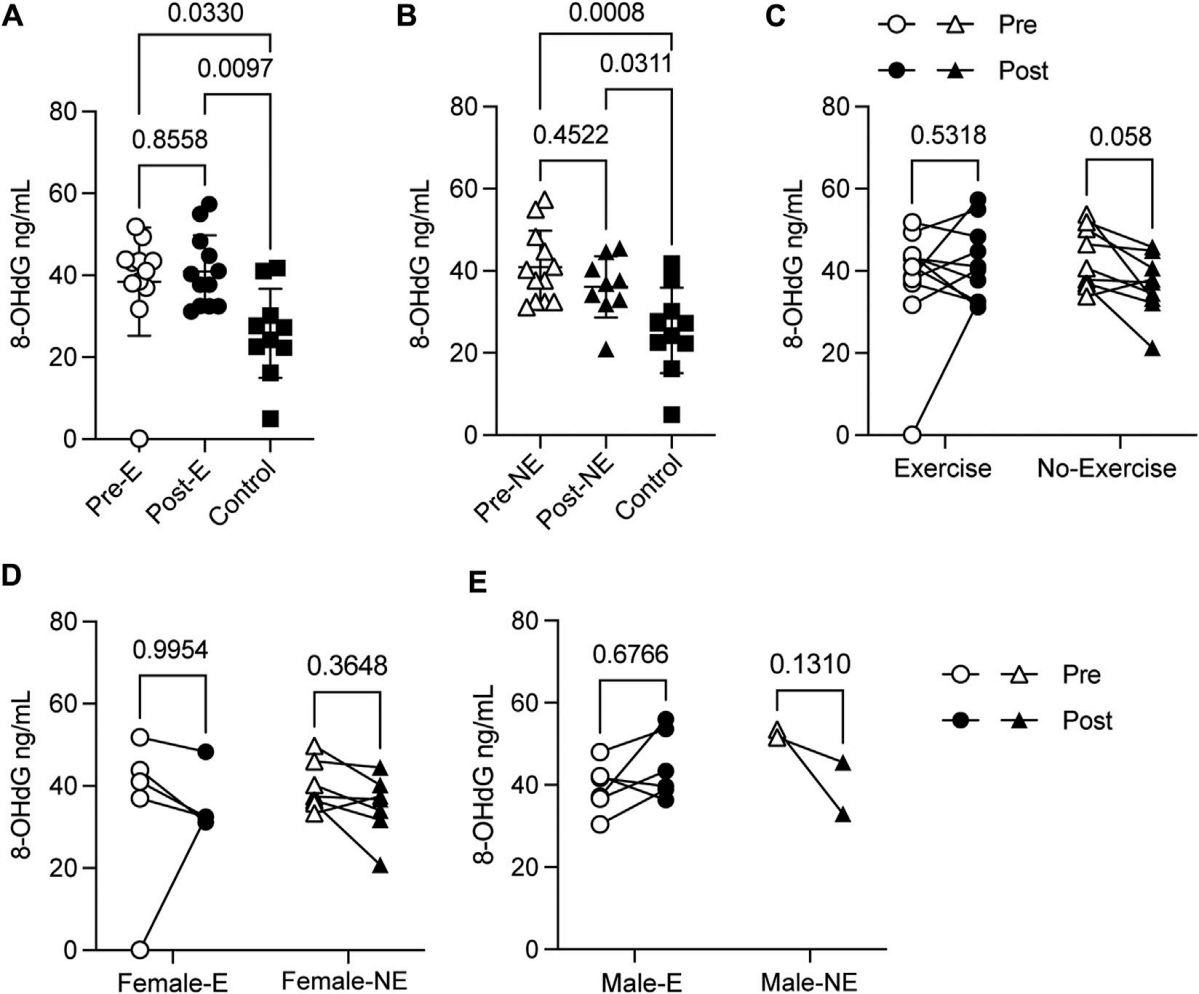
Concentration of 8-OHdG DNA in plasma samples measured by ELISA. **(A)** 8-OHdG DNA levels in PCC subjects before (Pre-E) and at the end (Post-E) of the exercise regimen compared to no-PCC controls. **(B)** 8-OHdG DNA concentration in no-exercise PCC subjects at the beginning (Pre-NE) and at the end (Post-NE) of the study period compared to no-PCC controls. **(C)** Paired analysis of pre- and post- 8-OHdG DNA levels within exercise and no-exercise PCC groups **(D, E)** Paired analysis of 8-OHdG DNA levels among female and male PCC subjects within exercise (-E) and no-exercise (-NE) groups. Statistical comparisons were carried out with Mann-Whitney’s test or the Wilcoxon test. 8-OHdG, 8-Hydroxy-2-Deoxyguanosine.

As, exercise is frequently linked to the production of free radicals and the induction of oxidative stress in an intensity-dependent manner (Powers and Jackson, 2008) we measured 2 markers of oxidative stress, namely, glutathione (GSH) and plasma 8-OHdG. Total glutathione levels markedly increased in the PBMCs of the intervention group after the exercise regimen whereas no such increase was observed in the no-exercise group at the corresponding post-time point (Figures 5A–C). When the analyses were carried out separately in females and males, the GSH levels showed significant increase in females with PCC following exercise intervention (Figure 5B *p* = 0.022). Such differences were not evident in males with PCC who were subject to a comparable exercise regimen (Figure 5C), or in females who did not receive any intervention (Figure 5B). In addition to GSH, we measured the levels of 8-OHdG, which is produced when the hydroxyl radical oxidizes guanine following oxidative damage of DNA. The 8-OHdG levels were significantly elevated in the PCC group when compared to the no-PCC group (Figures 6A, B). However, no significant differences in 8-OHdG levels were found between the exercise and non-exercise intervention groups (Figure 6C). Unlike the GSH levels, which increased significantly in the exercise group females, no appreciable changes were observed in the 8-OHdG levels in female or male PCC patients before or after exercise (Figures 6D, E). These findings indicate that exercise-induced oxidative stress response does not adversely affect the redox homeostasis in PCC patients.

### 3.6 Impact of the exercise intervention on the plasma proteome of PCC patients

**FIGURE 7:**
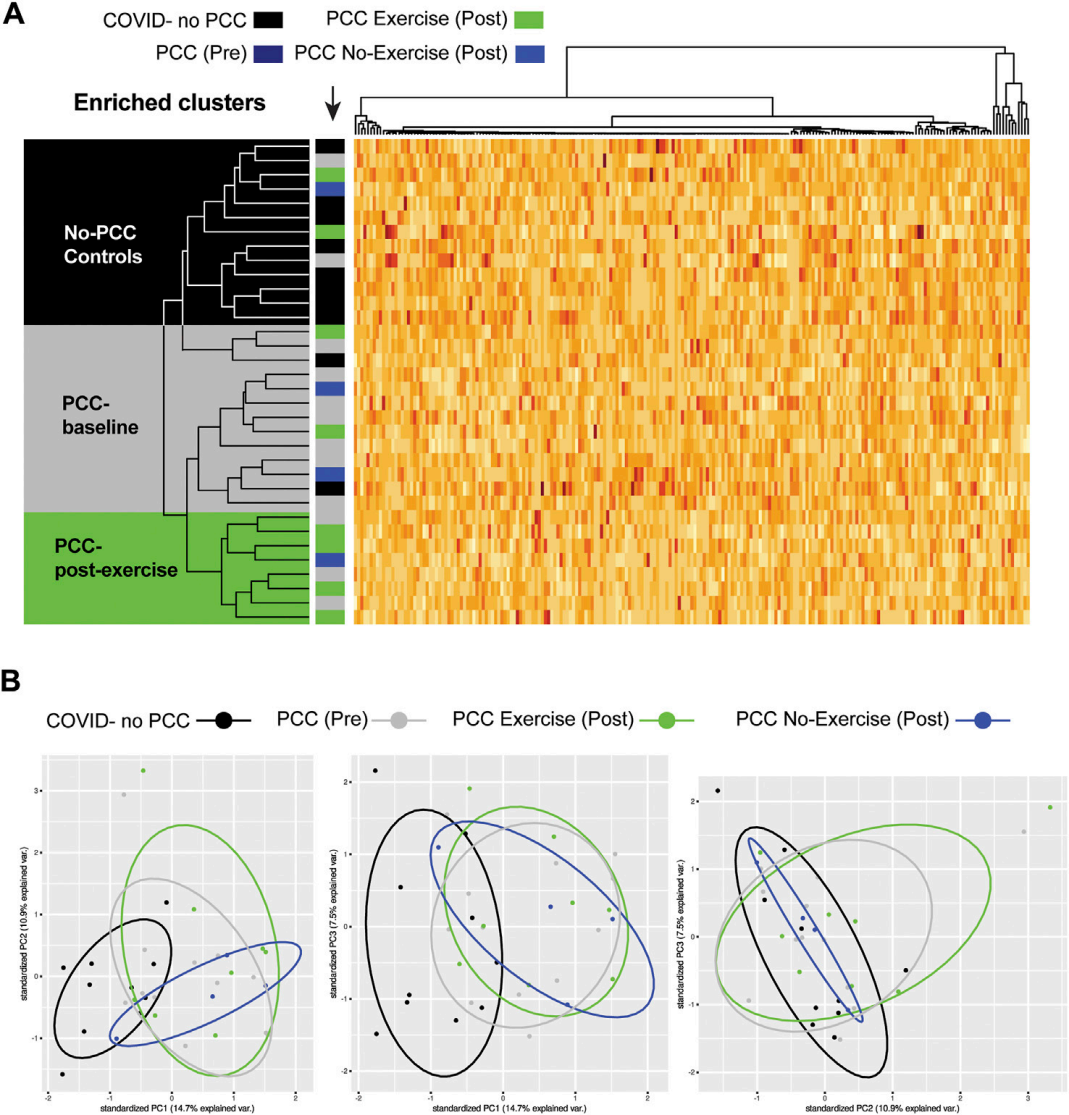
**(A)** Unsupervised clustering analysis showing segregation of the proteomic data of the PCC study groups. Most of the No-PCC controls cluster together (black). Most PCC samples collected at the beginning of the study cluster together at baseline (grey), whereas those who were subject to tailored exercise intervention clustered into a distinct group (green). **(B)** Principal component analysis (PCA).

To study global changes induced by the exercise regimen in PCC patients, we compared their plasma protein profiles before and after the intervention period in 22 of the 25 individuals shown in Table 2. All samples that clustered together on the heatmap and based on a cut off with a Pearson correlation ≥ 0.9875 were removed (non-movers). Interestingly, seven out of 10 paired samples (1/2 males and 5/8 females) in the no-exercise group (baseline and after 8 weeks of no intervention) did not show significant difference in the protein profile between pre- and post-condition. The filtering was done by doing an heatmap on all data. In the PCC group that went through the exercise training regimen, eight out of 12 paired samples showed significant differences between pre- and post-intervention (3/6 males and 5/6 females). Unsupervised clustering analysis of the proteomic data from PCC samples (both exercise and no-exercise groups) that showed significant differences at the baseline and at the end of the study period as well as the No-PCC group showed distinct clustering of these study groups (Figure 7A). Most of the No-PCC control samples cluster together and are distinct from the PCC group at baseline. PCC-post exercise group displayed a tendency to cluster separately. The no-PCC group also appeared to be distinct in the principal component analysis (PCA), whereas the PCC groups before and after exercise intervention did not show distinct segregation (Figure 7B).

**FIGURE 8:**
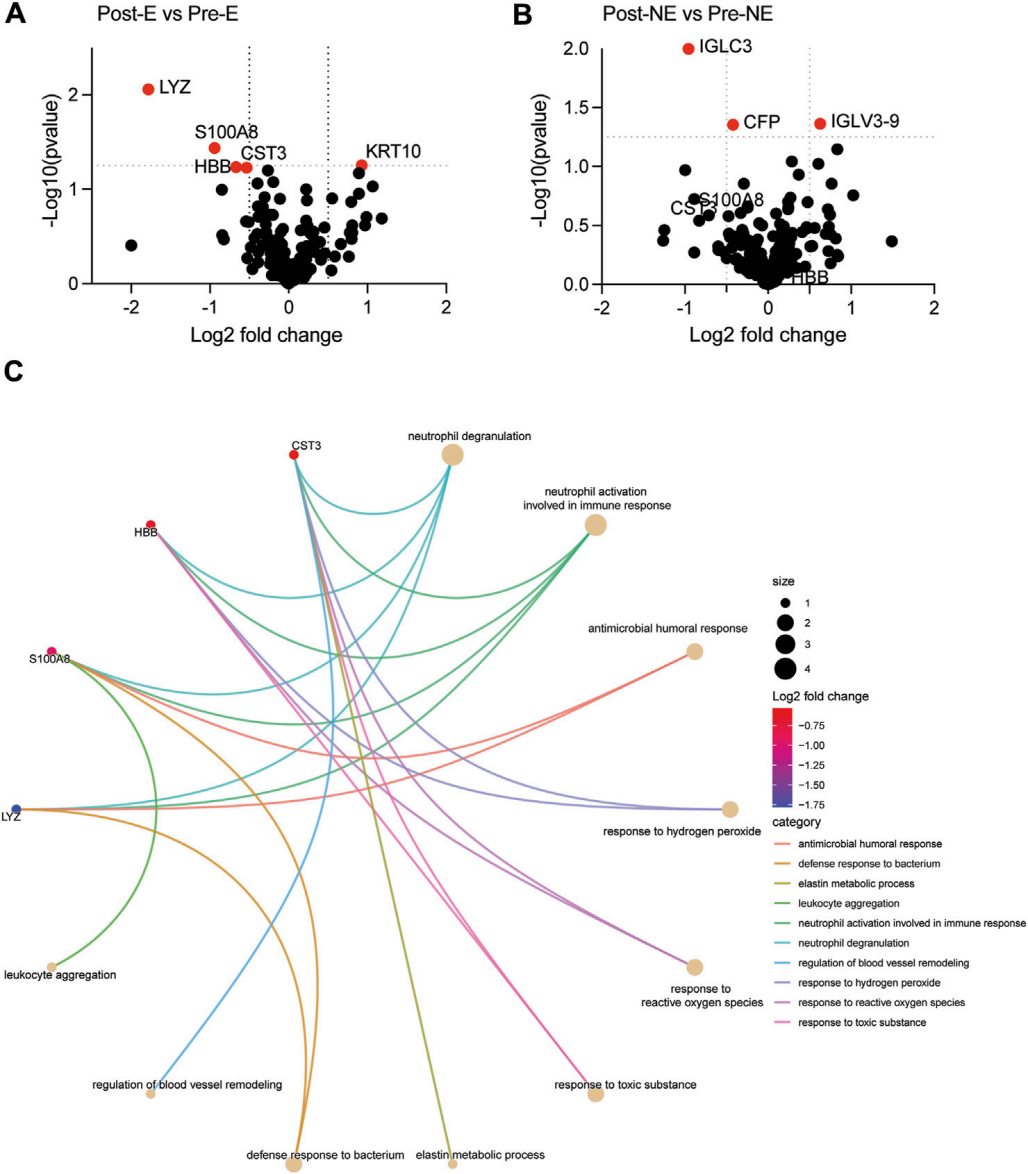
Volcano plot comparing the plasma proteome of the post-exercise group with that of the pre-exercise group (**A**; Post-E versus Pre-E), and the corresponding data within the no-exercise groups (**B**; Post-NE versus Pre-NE). Proteins that are signicantly modulated (fold change of ± 0.5 and *p*-value of 0.05, indicated by dotted line) are labelled are indicated in red color. **(C)** cnet plot of pathways identified to be downregulated in the exercise group.

The plasma proteomes of pre- and post-collection samples within the exercise and No-exercise groups were compared by volcano plots to identify proteins that are differentially modulated at the end of the study period (Figures 8A, B). Even though the plasma samples were obtained from the same individual within a period of 2 months, a few differentially expressed proteins were clearly identified. These candidate proteins include CST3, LYZ, S100A8 that showed at least a 1.5-fold reduction following exercise intervention but displayed no significant changes in the no-exercise group (Figures 8A, B; Supplementary Figure S2). Cnet analysis of proteins differentially regulated in the exercise group before and after the intervention implicated these three proteins in neutrophil activation and degranulation (Figure 8C). KRT10 was the only plasma protein that was significantly upregulated in the exercise group (Figure 8A). Paired analyses of the significantly modulated proteins in the pre- and post-regimen samples revealed marked downregulation of SA100A8 and upregulation of KRT10 that are close to the significance levels only within the exercise group, whereas the downregulation of CST3 and LYZ were not as markedly different, presumably due to the low sample number (Supplementary Figure S2).

Next, we evaluated the expression of CST3 and S100A8 by ELISA to determine whether these markers could be detected in the plasma samples by classical methods for objective monitoring of the impact of the exercise regimen. Plasma CST3 levels in both exercise and no-exercise PCC groups were significantly lower than in no-PCC controls, and there was no significant difference in the pre- and post-regimen time points in both groups (Supplementary Figures S3A, B). On the other hand, plasma S100A8 levels were not significantly different in both the PCC groups compared no-PCC controls, although a marked increase was observed in the no-exercise group at the end of the study period (Supplementary Figures S3C, D).

## 4 Discussion

To our knowledge, this is the first study that analyzes the effect of tailored exercise in COVID-19 afflicted individuals who exhibited PCC symptoms 1 year after clearing the infection, along with the impact of exercise intervention on systemic oxidative stress and plasma proteome. This study highlights two important observations: 1) moderate tailored exercise regimen aimed at enhancing muscular strength and endurance in PCC patients resulted in measurable improvements in physical capacity tests such as Bicep curl and STS-30, together with a beneficial tendency for HGS and 6-MWT. More importantly, (an elevated fatigue could have been an indicator of post-exertion malaise potentially induced from the previous training session) or other PCC symptoms. 2) These changes are associated with discernible modulation of systemic oxidative stress and the levels of certain plasma proteins implicated in inflammatory responses at the end of the 2 months of moderate intensity tailored exercise regimen.

### 4.1 Physical capacity and PASE

At the time our protocol was established, almost no literature existed regarding the variability physical capacity in patients with PCC with and without exercise. We therefore, chose tests that we have experience with (Marechal et al., 2019), and are frequently used in the functional community-dwelling elderly population for the following reasons: 1- on first appraisal, our PCC patients level of function appeared equivalent; 2- these tests are frequently used to measure exercise-induced functional improvements in aging adults, and 3- these tests are closely associated with daily functioning, which concurred with our overarching goal to help these patients be more functional in everyday life. Although the use of these tests in younger individuals could be debatable, the observed significant improvements in STS-30, biceps curl (time and interaction effects) and 6MWT (time effect) over 8 weeks support the absence of a ceiling effect. In addition, measured baseline values in PCC patients from our study are equivalent or even lower than the Senior Fitness Test reference values for the elderly (>60 years) men and women (Fournier et al., 2012) or the handgrip strength reference values (Wong, 2016) for Canadians men and women (>60 years), which reinforce the relevance of using this physical capacity methodology.

Our results show that the program had a positive effect on muscular strength and endurance. Indeed, we observed an improvement of approximately four repetitions in the exercise group for the STS-30, whereas the no-exercise group improved only by two repetitions. This is likely explained by an increased functional capacity of the lower limbs since the exercise program included exercises that specifically targeted the lower limbs (e.g., squats, lunges, hip abduction). Nevertheless, upper body was also significantly improved by 8 weeks of RT as shown by significant improvements in biceps curl. Handgrip strength is an indicator of muscle endurance and strength (Trosclair et al., 2011) but is also associated with mortality and cardiometabolic multimorbidity (Lu et al., 2022), suggesting that exercise can confer long term benefits to PCC patients.

Regarding cardiopulmonary function we observed that both exercise and no-exercise groups improved their performances at the 6MWT, even though it was not significantly different. First, it cannot be ruled out that a learning effect may have taken place. However, the presence of the control group corrects for this potential bias. Indeed, the exercise group increased its performance by almost the double compared to the no-exercise group. Interestingly, the minimal clinically important difference (MCID) was shown to be 14.0–30.5 m for the 6MWT (Bohannon and Crouch, 2017). However, most studies report a MCID of at least 25 m in patients with a pulmonary or cardiovascular condition. Interestingly, while the control group improved their performance of 24.72 m, barely reaching the MCID, the exercise group improved by 44.99 m during the 8-week intervention, largely exceeding the MCID. Since a longer duration of intervention would have likely resulted in statistically significant improvement, our findings support to pursue research with regards to the clinical utility of an exercise intervention to improve cardiorespiratory function in PCC patients.

The physical tests used in this study are part of the senior fitness test (Rikli and Jones, 2013) and were chosen, as mentioned above, in coherence with the anticipated level of physical capacity of the participants. Additionally, these tests were selected because the results could show a global portrait of lower and upper body strength and indicate a certain range of cardiorespiratory function. Also, these tests have the advantage of being easy to implement and could thus be used routinely in clinical settings. Despite their simplicity, these tests appear to be adequate to achieve measurable improvements in physical capacity that manifest within the short duration of 8 weeks. Our intervention regimen was based on a study conducted in 27 patients. Although our protocol differs from most (e.g.,: cardiovascular testing and 1 repetition-maximum (1RM) testing) (Stavrou et al., 2021; Tanguay et al., 2021; Jimeno-Almazan et al., 2022), our physical capacity testing had many similarities with one recent study (Yelin et al., 2023). The study by the group of Yelin et al. (2023) were similar to our study, comprising more women than men, with a mean age of 48.3 ± 10.2 years and a BMI of 28.8 ± 6.3 kg/m^2^. Comparison of commonly used physical capacity tests within both studies show that participants in our exercise group exceeded in performance after the very first visit, possibly reflecting the large variability of clinical manifestations in PCC patients. Smer and al (Smer et al., 2023). suggested the use of the 6MWT to assess exercise capacity, to start with less than 10 min of aerobic training daily, exercising for two to three non-consecutive sessions per week, 10-15 repetitions of RT exercises, for a total of 8–10 exercises, supervised and individualized sessions. Our protocol is in line with these recommendations and our exercise regimen seems to be appropriate for PCC patients, as supported by the measurable efficiency of this strategy.

The greater increase in physical activity level in the exercise group indicates a positive effect of the program on their daily functioning. The score also improved, although less dramatically, in the no-exercise group, which indicates that physical capacity improves over time in PCC patients. Still, our results suggest that an exercise intervention may support a faster return to pre-PCC levels. Although our results show no improvement in PCC symptoms in the exercise group, it is noteworthy that the questionnaire we used did not measure the severity of symptoms, which is obviously a limit of the study. It is thus possible that although still present, the severity has decreased such as suggested by our direct observations. This obviously needs to be further investigated. Nevertheless, it is important to mention that general fatigue did not worsen throughout the program, such as purported by some health professionals in the media. This shows that our exercise training program respected the participants’ physical capacity, and that physical exercise may be an efficient strategy to accelerate physical capacity recovery in LC patients, without inducing post-exertion malaise in those who never experienced it.

### 4.2 Exercise associated molecular changes in PCC patients

The tailored exercise regimen appears to have a positive effect on systemic oxidative stress in PCC patients, as shown in this study. One of the non-pharmacological intervention methods that decrease oxidative stress is moderate physical activity (Forman and Zhang, 2021). While intense physical activity increases oxidative stress in the immediate time points after physical exercise, moderate physical activity over prolonged periods of time reduces oxidative stress (El Assar et al., 2022). Glutathione is a major antioxidant peptide found in almost all cell types including red blood cells and is required to counter the oxidative stress (Forman et al., 2009). GSH is a tripeptide consisting of glycine, cysteine and glutamate where the active thiol group of the cysteine plays a major role in antioxidant responses. Reduced GSH (GSSG) levels inversely correlates to COVID-19 severity (Kumar et al., 2021). Decreased GSH level in brain tissues was associated with COVID-19 survivors who developed symptoms of depression and displayed differences in the volume of cerebral white matter hyperintensities that is associated with cognitive impairment (Poletti et al., 2022). However, in this study we did not measure all the parameters of pro- and anti-oxidant levels to assess the overall outcome of the effect of exercise on oxidative stress. Gender and sex have been reported to influence the different markers of oxidative stress (Martinez de Toda et al., 2023). Our observations indicate that in PCC, tailored exercise may decrease the oxidative stress as seen from the increase in the levels GSH in female PCC patients. Increased levels of GSH have been observed in women with hyperlipidemic type 2 diabetes (Darmian et al., 2022) while exercise-associated increase in GSH levels did not show significant differences between healthy men and women (Elosua et al., 2003; McAllister et al., 2022). One main difference between our study and the above-mentioned reports is the cellular source of GSH. In our study GSH levels were measured in the PBMCs while the other reports measured the GSH activity in the peripheral blood red blood cells. The concordance of these results suggests that oxidative stress can manifest at the systemic level in circulating blood cells and that physical exercise can cause a measurable reduction in the magnitude of oxidative stress conditions.

Since the beginning of the COVID-19 pandemic, oxidative stress has been linked to disease severity (Kumar et al., 2021). However, elevated oxidative stress is not unique to SARS-CoV-2, as various other viral infections also increase oxidative stress (Oda et al., 1989; Hennet et al., 1992; Palamara et al., 1995). Oxidative stress is also implicated in chronic inflammatory diseases and cancer (Forman and Zhang, 2021). Oxidative stress activates NRF2, which induces a transcriptional program of different proteins involved in attenuating the oxidative stress and maintaining cellular redox homeostasis (He et al., 2020). However, development of interventions targeting oxidative stress to ameliorate the underlying disease state are nascent and are not yet part of standard treatment (Forman and Zhang, 2021). Treatment with vitamin C (ascorbic acid), which can also reduce oxidative stress (Guaiquil et al., 2001), had beneficial effects in long COVID (Izzo et al., 2022). Reductions in inflammatory and oxidative stress markers was observed in a small group of athletes and non-athletes with long COVID following hyperbaric oxygen therapy over a period of 4 months with a concomitant decrease in chronic fatigue (Mrakic-Sposta et al., 2023).

Various groups have observed changes in the inflammatory and coagulation pathways in the plasma proteome of long COVID patients (Gu et al., 2023; Lopez-Hernandez et al., 2023; Woodruff et al., 2023). To our knowledge, our study is the first to investigate changes to plasma proteomics following exercise intervention in PCC patients. The exercise regimen was administered for only 8 weeks, which is a relative short period of intervention considering the chronicity of PCC. Even though the effect of exercise was not distinguishable among the two PCC groups, which segregated distinctly from the no-PCC group, certain inflammatory markers, notably S100A8 and CST3 showed a downward trend in paired analysis of the pre- and post-exercise group. As these two proteins are implicated in neutrophil activation and degranulation pathways and have been previously implicated in COVID-19 (Shrivastava et al., 2021; Gupta et al., 2022; Holms, 2022), their reduction following exercise regimen suggests a declining level of inflammatory conditions that could contribute to the improved PASE score in the post-exercise PCC group.

Our efforts to use an ELISA based assay to detect changes in plasma SA100A8 and CST3 protein levels to monitor reduced inflammatory conditions and oxidative stress were not encouraging, as the paired analyses of ELISA-based quantification did not correlate with similar analysis of the mass spectrometry data. Whereas mass spectrometry detects unique linear peptide sequences from intact and fragmented proteins, ELISA assays mostly rely on detection of restricted epitopes on the same protein. Hence, it is possible that the ELISA assays are unable to fully capture the real abundance of SA100A8 and CST3 (otherwise detected as peptides by mass spectrometry) contributing to the apparent discrepancies between the two assays. One approach to circumvent this issue would be to use a targeted mass spectrometry-based quantification of the most abundant peptide sequences of these proteins (Liebler and Zimmerman, 2013; Renuse et al., 2021) to monitor improvement of systemic inflammation.

## 5 Conclusion

Moderate tailored exercise regimen aimed at enhancing muscular strength and endurance is safe in PCC patients and was not associated with fatigue or other signs of post-exertion malaise, which directed against exercise in all PCC patients by the clinical milieu. These changes can be followed at the systemic level by analyzing oxidative stress markers and plasma proteome.

## 6 Limitations and strengths of the study and future research

Two obvious limitations of this study are the small sample size, the short duration of the exercise intervention and absence of long-term follow up of the participants. Firstly, the small sample size results from the difficulty encountered in recruitment when the study was conducted from 11 March 2022, to 18 April 2023 and the unwillingness of most PCC patients to participate in such exploratory study. Out of the 340 PCC patients who were initially contacted, only 32 participants consented to participate in this study. One reason for this lack of enthusiasm to participate in exercise-based clinical trials could be the discussion in the social networks and conventional media on post-exertion malaise and contradictory messaging about physical exercise. After exclusions and dropouts, we could complete the study on 25 participants with some imbalance between men and women among exercise and no-exercise groups. It cannot be ruled out that some sex effect contributed in part to the results, which deserved further investigation. Besides, the continuous spectrum of PCC symptoms and the inability to sub-group patients based on symptoms contributed to the heterogeneity in the recruited population. In the same line, our results cannot be applied to PCC patients who have experienced post-exertion malaise since they were not included in the study. Secondly, the duration of the exercise regimen used in our study was designed based on the data available at that time (as discussed earlier) while keeping with the possibility that a longer study might reduce compliance. In addition to the aforementioned limits, we have to reiterate that a more robust trial addressing other methodological concerns such as the use of 6MWT to estimate cardiorespiratory function or the lack of symptom severity measurement is needed. Despite these caveats, our study provides evidence-based information that a tailored exercise program is feasible and can have a favorable outcome in PCC. Our study also provides the first evidence that the physiological effects of tailored low-to-moderate intensity exercise can be monitored by minimally invasive methods of analyses of the associated biological processes in the peripheral blood. Glutathione (GSH) is an important endogenous antioxidant which neutralizes reactive oxygen species (ROS) and other free radicals, protecting cells from oxidative damage and cellular component like DNA, proteins. Decreased levels of GSH are associated with increased oxidative stress which observed in COVID-19 research. We focused on the major antioxidant GSH to understand the DNA damage (8-oHdG) level. Measurement of different parameters related to oxidative stress would have provided a complete picture. The interpretation of the various biochemical and immunological results will become more informative with a larger study population and inclusion of infected individuals who do not develop PCC.

## Data Availability

The datasets presented in this study can be found in online repositories. The names of the repository/repositories and accession number(s) can be found below: https://www.ebi.ac.uk/pride/archive/, PXD047623.

## References

[B1] Agha N. H. Baker F. L. Kunz H. E. Graff R. Azadan R. Dolan C. (2018). Vigorous exercise mobilizes CD34+ hematopoietic stem cells to peripheral blood via the β2-adrenergic receptor. Brain Behav. Immun. 68, 66–75. 10.1016/j.bbi.2017.10.001 29017969 PMC6980177

[B2] Ahmadi Hekmatikar A. H. Ferreira Junior J. B. Shahrbanian S. Suzuki K. (2022). Functional and psychological changes after exercise training in post-COVID-19 patients discharged from the hospital: a PRISMA-compliant systematic review. Int. J. Environ. Res. Public Health 19, 2290. 10.3390/ijerph19042290 35206483 PMC8871540

[B3] Akerboom T. P. Sies H. (1981). Assay of glutathione, glutathione disulfide, and glutathione mixed disulfides in biological samples. Methods Enzymol. 77, 373–382. 10.1016/s0076-6879(81)77050-2 7329314

[B4] Batatinha H. A. P. Kruger K. Rosa Neto J. C. (2020). Thromboinflammation and COVID-19: the role of exercise in the prevention and treatment. Front. Cardiovasc Med. 7, 582824. 10.3389/fcvm.2020.582824 33392268 PMC7775570

[B5] Bohannon R. W. Crouch R. (2017). Minimal clinically important difference for change in 6-minute walk test distance of adults with pathology: a systematic review. J. Eval. Clin. Pract. 23, 377–381. 10.1111/jep.12629 27592691

[B6] Bonetto V. Pasetto L. Lisi I. Carbonara M. Zangari R. Ferrari E. (2022). Markers of blood-brain barrier disruption increase early and persistently in COVID-19 patients with neurological manifestations. Front. Immunol. 13, 1070379. 10.3389/fimmu.2022.1070379 36591311 PMC9798841

[B7] Borg G. A. (1982). Psychophysical bases of perceived exertion. Med. Sci. Sports Exerc 14, 377–381. 10.1249/00005768-198205000-00012 7154893

[B8] Darmian M. A. Hoseini R. Amiri E. Golshani S. (2022). Downregulated hs-CRP and MAD, upregulated GSH and TAC, and improved metabolic status following combined exercise and turmeric supplementation: a clinical trial in middle-aged women with hyperlipidemic type 2 diabetes. J. Diabetes Metab. Disord. 21, 275–283. 10.1007/s40200-022-00970-z 35106289 PMC8795726

[B9] Davis H. E. Assaf G. S. McCorkell L. Wei H. Low R. J. Re'em Y. (2021). Characterizing long COVID in an international cohort: 7 months of symptoms and their impact. EClinicalMedicine 38, 101019. 10.1016/j.eclinm.2021.101019 34308300 PMC8280690

[B10] El Assar M. Alvarez-Bustos A. Sosa P. Angulo J. Rodriguez-Manas L. (2022). Effect of physical activity/exercise on oxidative stress and inflammation in muscle and vascular aging. Int. J. Mol. Sci. 23, 8713. 10.3390/ijms23158713 35955849 PMC9369066

[B11] Elosua R. Molina L. Fito M. Arquer A. Sanchez-Quesada J. L. Covas M. I. (2003). Response of oxidative stress biomarkers to a 16-week aerobic physical activity program, and to acute physical activity, in healthy young men and women. Atherosclerosis 167, 327–334. 10.1016/s0021-9150(03)00018-2 12818416

[B12] Fernandez-Lazaro D. Gallego-Gallego D. Corchete L. A. Fernandez Zoppino D. Gonzalez-Bernal J. J. Garcia Gomez B. (2021). Inspiratory muscle training program using the PowerBreath((R)): does it have ergogenic potential for respiratory and/or athletic performance? A systematic review with meta-analysis. Int. J. Environ. Res. Public Health 18, 6703. 10.3390/ijerph18136703 34206354 PMC8297193

[B13] Fernandez-Lazaro D. Santamaria G. Sanchez-Serrano N. Lantaron Caeiro E. Seco-Calvo J. (2022). Efficacy of therapeutic exercise in reversing decreased strength, impaired respiratory function, decreased physical fitness, and decreased quality of life caused by the post-COVID-19 syndrome. Viruses 14, 2797. 10.3390/v14122797 36560801 PMC9784943

[B14] Forman H. J. Zhang H. (2021). Targeting oxidative stress in disease: promise and limitations of antioxidant therapy. Nat. Rev. Drug Discov. 20, 689–709. 10.1038/s41573-021-00233-1 34194012 PMC8243062

[B15] Forman H. J. Zhang H. Rinna A. (2009). Glutathione: overview of its protective roles, measurement, and biosynthesis. Mol. Asp. Med. 30, 1–12. 10.1016/j.mam.2008.08.006 PMC2696075 18796312

[B16] Fournier J. Vuillemin A. Le Cren F. (2012). Mesure de la condition physique chez les personnes âgées. Évaluation de la condition physique des seniors: adaptation française de la batterie américaine « Senior Fitness Test ». Sci. Sports 27, 254–259. 10.1016/j.scispo.2012.07.005

[B17] Frion J. Meller A. Marbach G. Levesque D. Roucou X. Boisvert F. M. (2023). CRISPR/Cas9-mediated knockout of the ubiquitin variant UbKEKS reveals a role in regulating nucleolar structures and composition. Biol. Open 12, bio059984. 10.1242/bio.059984 37670689 PMC10537958

[B18] Global Burden of Disease Long C. C. Wulf Hanson S. Abbafati C. Aerts J. G. Al-Aly Z. Ashbaugh C. (2022). Estimated global proportions of individuals with persistent fatigue, cognitive, and respiratory symptom clusters following symptomatic COVID-19 in 2020 and 2021. JAMA 328, 1604–1615. 10.1001/jama.2022.18931 36215063 PMC9552043

[B19] Graff R. M. Kunz H. E. Agha N. H. Baker F. L. Laughlin M. Bigley A. B. (2018). β2-Adrenergic receptor signaling mediates the preferential mobilization of differentiated subsets of CD8+ T-cells, NK-cells and non-classical monocytes in response to acute exercise in humans. Brain Behav. Immun. 74, 143–153. 10.1016/j.bbi.2018.08.017 30172948 PMC12977291

[B20] Gu X. Wang S. Zhang W. Li C. Guo L. Wang Z. (2023). Probing long COVID through a proteomic lens: a comprehensive two-year longitudinal cohort study of hospitalised survivors. EBioMedicine 98, 104851. 10.1016/j.ebiom.2023.104851 37924708 PMC10660018

[B21] Guaiquil V. H. Vera J. C. Golde D. W. (2001). Mechanism of vitamin C inhibition of cell death induced by oxidative stress in glutathione-depleted HL-60 cells. J. Biol. Chem. 276, 40955–40961. 10.1074/jbc.M106878200 11533037

[B22] Guo Y. Wang S. Chao X. Li D. Wang Y. Guo Q. (2022). Multi-omics studies reveal ameliorating effects of physical exercise on neurodegenerative diseases. Front. Aging Neurosci. 14, 1026688. 10.3389/fnagi.2022.1026688 36389059 PMC9659972

[B23] Gupta A. Al-Tamimi A. O. Halwani R. Alsaidi H. Kannan M. Ahmad F. (2022). Lipocalin-2, S100A8/A9, and cystatin C: potential predictive biomarkers of cardiovascular complications in COVID-19. Exp. Biol. Med. (Maywood) 247, 1205–1213. 10.1177/15353702221091990 35466734 PMC9379606

[B24] He F. Ru X. Wen T. (2020). NRF2, a transcription factor for stress response and beyond. Int. J. Mol. Sci. 21, 4777. 10.3390/ijms21134777 32640524 PMC7369905

[B25] Hennet T. Peterhans E. Stocker R. (1992). Alterations in antioxidant defences in lung and liver of mice infected with influenza A virus. J. Gen. Virol. 73 (Pt 1), 39–46. 10.1099/0022-1317-73-1-39 1530963

[B26] Holms R. D. (2022). Long COVID (PASC) is maintained by a self-sustaining pro-inflammatory TLR4/RAGE-loop of S100a8/A9 > TLR4/RAGE signalling, inducing chronic expression of IL-1b, IL-6 and TNFa: anti-inflammatory ezrin peptides as potential therapy. Immuno 2, 512–533. 10.3390/immuno2030033

[B27] Huang C. Wang Y. Li X. Ren L. Zhao J. Hu Y. (2020). Clinical features of patients infected with 2019 novel coronavirus in Wuhan, China. Lancet 395, 497–506. 10.1016/S0140-6736(20)30183-5 31986264 PMC7159299

[B28] Humphreys H. Kilby L. Kudiersky N. Copeland R. (2021). Long COVID and the role of physical activity: a qualitative study. BMJ Open 11, e047632. 10.1136/bmjopen-2020-047632 PMC7948149 33692189

[B29] Izzo R. Trimarco V. Mone P. Aloe T. Capra Marzani M. Diana A. (2022). Combining L-Arginine with vitamin C improves long-COVID symptoms: the LINCOLN Survey. Pharmacol. Res. 183, 106360. 10.1016/j.phrs.2022.106360 35868478 PMC9295384

[B30] Jason L. A. Dorri J. A. (2022). ME/CFS and post-exertional malaise among patients with long COVID. Neurol. Int. 15, 1–11. 10.3390/neurolint15010001 36648965 PMC9844405

[B31] Jimeno-Almazan A. Buendia-Romero A. Martinez-Cava A. Franco-Lopez F. Sanchez-Alcaraz B. J. Courel-Ibanez J. (2023). Effects of a concurrent training, respiratory muscle exercise, and self-management recommendations on recovery from post-COVID-19 conditions: the RECOVE trial. J. Appl. Physiol. (1985) 134, 95–104. 10.1152/japplphysiol.00489.2022 36476156 PMC9829459

[B32] Jimeno-Almazan A. Franco-Lopez F. Buendia-Romero A. Martinez-Cava A. Sanchez-Agar J. A. Sanchez-Alcaraz Martinez B. J. (2022). Rehabilitation for post-COVID-19 condition through a supervised exercise intervention: a randomized controlled trial. Scand. J. Med. Sci. Sports 32, 1791–1801. 10.1111/sms.14240 36111386 PMC9538729

[B33] Kamal M. Abo Omirah M. Hussein A. Saeed H. (2020). Assessment and characterisation of post-COVID-19 manifestations. Int. J. Clin. Pract. 75, e13746. 10.1111/ijcp.13746 32991035 PMC7536922

[B34] Kumar P. Osahon O. Vides D. B. Hanania N. Minard C. G. Sekhar R. V. (2021). Severe glutathione deficiency, oxidative stress and oxidant damage in adults hospitalized with COVID-19: implications for GlyNAC (Glycine and N-acetylcysteine) supplementation. Antioxidants (Basel) 11, 50. 10.3390/antiox11010050 35052554 PMC8773164

[B35] Liebler D. C. Zimmerman L. J. (2013). Targeted quantitation of proteins by mass spectrometry. Biochemistry 52, 3797–3806. 10.1021/bi400110b 23517332 PMC3674507

[B36] Limoges M. A. Lortie A. Demontier E. Quenum A. J. I. Lessard F. Drouin Z. (2023a). SARS-CoV-2 mRNA vaccine-induced immune responses in rheumatoid arthritis. J. Leukoc. Biol. 114, 358–367. 10.1093/jleuko/qiad086 37478373 PMC10533224

[B37] Limoges M. A. Quenum A. J. I. Chowdhury M. M. H. Rexhepi F. Namvarpour M. Akbari S. A. (2023b). SARS-CoV-2 spike antigen-specific B cell and antibody responses in pre-vaccination period COVID-19 convalescent males and females with or without post-covid condition. Front. Immunol. 14, 1223936. 10.3389/fimmu.2023.1223936 37809081 PMC10551145

[B38] Lopez-Hernandez Y. Monarrez-Espino J. Lopez D. A. G. Zheng J. Borrego J. C. Torres-Calzada C. (2023). The plasma metabolome of long COVID patients two years after infection. Sci. Rep. 13, 12420. 10.1038/s41598-023-39049-x 37528111 PMC10394026

[B39] Lu Y. Li G. Ferrari P. Freisling H. Qiao Y. Wu L. (2022). Associations of handgrip strength with morbidity and all-cause mortality of cardiometabolic multimorbidity. BMC Med. 20, 191. 10.1186/s12916-022-02389-y 35655218 PMC9164350

[B40] Marechal R. Fontvieille A. Parent-Roberge H. Fulop T. Riesco E. Pavic M. (2019). Effect of a mixed-exercise program on physical capacity and sedentary behavior in older adults during cancer treatments. Aging Clin. Exp. Res. 31, 1583–1589. 10.1007/s40520-018-1097-4 30600490

[B41] Martinez de Toda I. Gonzalez-Sanchez M. Diaz-Del Cerro E. Valera G. Carracedo J. Guerra-Perez N. (2023). Sex differences in markers of oxidation and inflammation. Implications for ageing. Mech. Ageing Dev. 211, 111797. 10.1016/j.mad.2023.111797 36868323

[B42] McAllister M. J. Steadman K. S. Renteria L. I. Case M. J. Butawan M. B. Bloomer R. J. (2022). Acute resistance exercise reduces postprandial lipemia and oxidative stress in resistance-trained men. J. Strength Cond. Res. 36, 2139–2146. 10.1519/JSC.0000000000003831 33009352

[B43] Meier F. Brunner A. D. Frank M. Ha A. Bludau I. Voytik E. (2020). diaPASEF: parallel accumulation-serial fragmentation combined with data-independent acquisition. Nat. Methods 17, 1229–1236. 10.1038/s41592-020-00998-0 33257825

[B44] Mrakic-Sposta S. Vezzoli A. Garetto G. Paganini M. Camporesi E. Giacon T. A. (2023). Hyperbaric oxygen therapy counters oxidative stress/inflammation-driven symptoms in long COVID-19 patients: preliminary outcomes. Metabolites 13, 1032. 10.3390/metabo13101032 37887357 PMC10608857

[B45] Nalbandian A. Sehgal K. Gupta A. Madhavan M. V. McGroder C. Stevens J. S. (2021). Post-acute COVID-19 syndrome. Nat. Med. 27, 601–615. 10.1038/s41591-021-01283-z 33753937 PMC8893149

[B46] Nieman D. C. (2021). Exercise is medicine for immune function: implication for COVID-19. Curr. Sports Med. Rep. 20, 395–401. 10.1249/JSR.0000000000000867 34357885

[B47] Nieman D. C. Sakaguchi C. A. (2022). Physical activity lowers the risk for acute respiratory infections: time for recognition. J. Sport Health Sci. 11, 648–655. 10.1016/j.jshs.2022.08.002 35995362 PMC9391085

[B48] Norweg A. Yao L. Barbuto S. Nordvig A. S. Tarpey T. Collins E. (2023). Exercise intolerance associated with impaired oxygen extraction in patients with long COVID. Respir. Physiol. Neurobiol. 313, 104062. 10.1016/j.resp.2023.104062 37076024 PMC10108551

[B49] Oda T. Akaike T. Hamamoto T. Suzuki F. Hirano T. Maeda H. (1989). Oxygen radicals in influenza-induced pathogenesis and treatment with pyran polymer-conjugated SOD. Science 244, 974–976. 10.1126/science.2543070 2543070

[B50] Palamara A. T. Perno C. F. Ciriolo M. R. Dini L. Balestra E. D'Agostini C. (1995). Evidence for antiviral activity of glutathione: *in vitro* inhibition of herpes simplex virus type 1 replication. Antivir. Res. 27, 237–253. 10.1016/0166-3542(95)00008-a 8540746

[B51] Poletti S. Paolini M. Mazza M. G. Palladini M. Furlan R. Querini P. R. (2022). Lower levels of glutathione in the anterior cingulate cortex associate with depressive symptoms and white matter hyperintensities in COVID-19 survivors. Eur. Neuropsychopharmacol. 61, 71–77. 10.1016/j.euroneuro.2022.06.008 35810586 PMC9239982

[B52] Powers S. K. Jackson M. J. (2008). Exercise-induced oxidative stress: cellular mechanisms and impact on muscle force production. Physiol. Rev. 88, 1243–1276. 10.1152/physrev.00031.2007 18923182 PMC2909187

[B53] Reiss S. Baxter A. E. Cirelli K. M. Dan J. M. Morou A. Daigneault A. (2017). Comparative analysis of activation induced marker (AIM) assays for sensitive identification of antigen-specific CD4 T cells. PLoS One 12, e0186998. 10.1371/journal.pone.0186998 29065175 PMC5655442

[B54] Renuse S. Vanderboom P. M. Maus A. D. Kemp J. V. Gurtner K. M. Madugundu A. K. (2021). A mass spectrometry-based targeted assay for detection of SARS-CoV-2 antigen from clinical specimens. EBioMedicine 69, 103465. 10.1016/j.ebiom.2021.103465 34229274 PMC8253671

[B55] Rikli R. E. Jones C. J. (2013). Development and validation of criterion-referenced clinically relevant fitness standards for maintaining physical independence in later years. Gerontologist 53, 255–267. 10.1093/geront/gns071 22613940

[B56] Sette A. Sidney J. Crotty S. T. (2023). T cell responses to SARS-CoV-2. Annu. Rev. Immunol. 41, 343–373. 10.1146/annurev-immunol-101721-061120 36750314

[B57] Shrivastava S. Chelluboina S. Jedge P. Doke P. Palkar S. Mishra A. C. (2021). Elevated levels of neutrophil activated proteins, alpha-defensins (DEFA1), calprotectin (S100a8/A9) and myeloperoxidase (MPO) are associated with disease severity in COVID-19 patients. Front. Cell Infect. Microbiol. 11, 751232. 10.3389/fcimb.2021.751232 34746027 PMC8566808

[B58] Smer A. Squires R. W. Bonikowske A. R. Allison T. G. Mainville R. N. Williams M. A. (2023). Cardiac complications of COVID-19 infection and the role of physical activity. J. Cardiopulm. Rehabil. Prev. 43, 8–14. 10.1097/HCR.0000000000000701 35839441 PMC9828583

[B59] Soril L. J. J. Damant R. W. Lam G. Y. Smith M. P. Weatherald J. Bourbeau J. (2022). The effectiveness of pulmonary rehabilitation for Post-COVID symptoms: a rapid review of the literature. Respir. Med. 195, 106782. 10.1016/j.rmed.2022.106782 35272262 PMC8887973

[B60] Stavrou V. T. Tourlakopoulos K. N. Vavougios G. D. Papayianni E. Kiribesi K. Maggoutas S. (2021). Eight weeks unsupervised pulmonary rehabilitation in previously hospitalized of SARS-CoV-2 infection. J. Pers. Med. 11, 806. 10.3390/jpm11080806 34442450 PMC8399744

[B61] Tanguay P. Marquis N. Gaboury I. Kairy D. Touchette M. Tousignant M. (2021). Telerehabilitation for post-hospitalized COVID-19 patients: a proof-of-concept study during a pandemic. Int. J. Telerehabil 13, e6383. 10.5195/ijt.2021.6383 34345354 PMC8287730

[B62] Trosclair D. Bellar D. Judge L. W. Smith J. Mazerat N. Brignac A. (2011). Hand-grip strength as a predictor of muscular strength and endurance. J. Strength and Cond. Res. 25, S99. 10.1097/01.jsc.0000395736.42557.bc

[B63] Twomey R. DeMars J. Franklin K. Culos-Reed S. N. Weatherald J. Wrightson J. G. (2022). Chronic fatigue and postexertional malaise in people living with long COVID: an observational study. Phys. Ther. 102, pzac005. 10.1093/ptj/pzac005 35079817 PMC9383197

[B64] Washburn R. A. Smith K. W. Jette A. M. Janney C. A. (1993). The physical activity scale for the elderly (PASE): development and evaluation. J. Clin. Epidemiol. 46, 153–162. 10.1016/0895-4356(93)90053-4 8437031

[B65] WHO (2021). A clinical case definition of post COVID-19 condition by a Delphi consensus. Available at: https://www.who.int/publications-detail-redirect/WHO-2019-nCoV-Post_COVID-19_condition-Clinical_case_definition-2021.1. 10.1016/S1473-3099(21)00703-9 PMC8691845 34951953

[B66] Wong S. L. (2016). Reduced muscular strength among Canadians aged 60 to 79: Canadian Health Measures Survey, 2007 to 2013. Health Rep. 27, 11–17. 27759871

[B67] Woodruff M. C. Bonham K. S. Anam F. A. Walker T. A. Faliti C. E. Ishii Y. (2023). Chronic inflammation, neutrophil activity, and autoreactivity splits long COVID. Nat. Commun. 14, 4201. 10.1038/s41467-023-40012-7 37452024 PMC10349085

[B68] Wright J. Astill S. L. Sivan M. (2022). The relationship between physical activity and long COVID: a cross-sectional study. Int. J. Environ. Res. Public Health 19, 5093. 10.3390/ijerph19095093 35564488 PMC9105041

[B69] Wu Z. McGoogan J. M. (2020). Characteristics of and important lessons from the coronavirus disease 2019 (COVID-19) outbreak in China: summary of a report of 72314 cases from the Chinese center for disease control and prevention. JAMA 323, 1239–1242. 10.1001/jama.2020.2648 32091533

[B70] Yelin D. Levi R. Babu C. Moshe R. Shitenberg D. Atamna A. (2023). Assessment of exercise capacity of individuals with long COVID: a cross-sectional study. Isr. Med. Assoc. J. 25, 83–87. 36841973

