# Supplementary material for: Impact of a tailored exercise regimen on physical capacity and plasma proteome profile in post-COVID-19 condition
Source: Front Physiol. 2024 Aug 21;15:1416639. doi: 10.3389/fphys.2024.1416639 (PMC11371593; doi:10.3389/fphys.2024.1416639)
Supplement: Supplementary file 1 [file Table1.docx]

**Supplementary Table 1.** Antibodies and reagents for flowcytometry.

| **Marker** | **antibody-conjugate** | | **clone** | **catalog #** | | **Source** | | **dilution** |  |  |  |  |
| --- | --- | --- | --- | --- | --- | --- | --- | --- | --- | --- | --- | --- |
| CD3 | AF700 Anti-human CD3 | | UCHT1 | 300424 | | BioLegend | | 1:300 |  |  |  |  |
| CD14 | AF700 Anti-human CD14 | | 63D3 | 367114 | | BioLegend | | 1:300 |  |  |  |  |
| CD56 | AF700 Anti-human CD56 | | HCD56 | 318316 | | BioLegend | | 1:300 |  |  |  |  |
| CD19 | APC-Cy7 Anti-human CD19 | | HIB19 | 302218 | | BioLegend | | 1:300 |  |  |  |  |
| CD27 | BV605 Anti-human CD27 | | O323 | 302830 | | BioLegend | | 1:300 |  |  |  |  |
| CD20 | PE-Cy7 Anti-human CD20 | | 2H7 | 302312 | | BioLegend | | 1:300 |  |  |  |  |
| IgD | BV785 Anti-human IgD | | IA6-2 | 348242 | | BioLegend | | 1:300 |  |  |  |  |
|  | APC Streptavidin | |  | 405207 | | BioLegend | | Protein:Strep = 4:1 |  |  |  |  |
|  | FITC streptavidin | |  | 405202 | | BioLegend | | Protein:Strep = 4:1 |  |  |  |  |
| CD3 | APC/Cy7 Anti-human CD3 | | HIT3a | 300318 | | BioLegend | | 1:400 |  |  |  |  |
| CD4 | BV605 anti-human CD4 | | SK3 | 566908 | | BD Bioscicences | | 1:400 |  |  |  |  |
| CD8a | BV510 Anti-human CD8a | | HIT8a | 300934 | | BioLegend | | 1:400 |  |  |  |  |
| CD14 | AF700 Anti-human CD14 | | 63D3 | 367114 | | BioLegend | | 1:400 |  |  |  |  |
| CD19 | AF700 Anti-human CD19 | | SJ25C1 | 363034 | | BioLegend | | 1:400 |  |  |  |  |
| CD25 | BV785 Anti-human CD25 | | BC96 | 302638 | | BioLegend | | 1:400 |  |  |  |  |
| CD56 | BV700 anti-human CD56 | | B159 | 566400 | | BD Biosciences | | 1:300 |  |  |  |  |
| CD69 | PE-Dazzle 594 Anti-human CD69 | | FN50 | 310941 | | BioLegend | | 1:400 |  |  |  |  |
| SARS-CoV-2 spike peptide pool | PepTivator SARS-CoV-2 Prot_S | |  | 130-126-700 | | Miltenyi Biotec | |  | | |  |  |
| wt-RBD | SARS-CoV-2 Spike RBD, His Tag (B.1.1.529/Omicron) | |  | SPD-C522e | | Acrobiosystems | |  | | |  |  |
|  | |  | | |  | |  | | |  | |  |

**
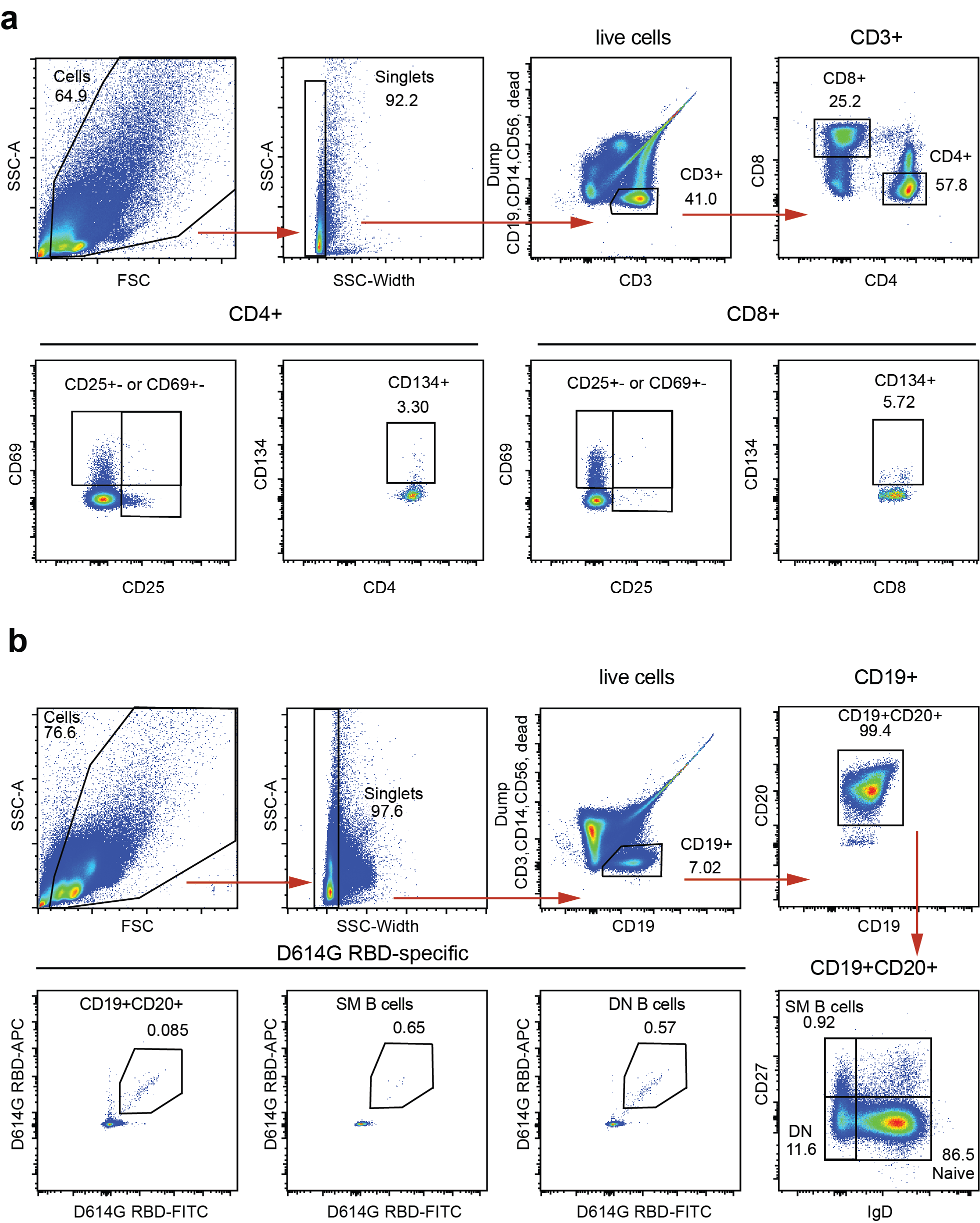
**

**Supplementary Figure S1**. Gating strategy to quantify spike peptides specific T cells and D614G RBD-specific B cells. a) PBMCs activated with spike peptides were labelled with T cell markers (CD3, CD4, CD8, CD25, CD69 and CD134) and AF-700 conjugated antibodies for B cells, monocytes and NK (dump channel). b) PBMCs were labelled with B cell markers (CD19, CD20, CD27 and IgD), AF-700 conjugated antibodies for T cells, monocytes and NK (dump channel), D614G-RBD-FITC and D614G-RBD-APC. APC and FITC double positive cells are considered D614G-RBD positive. CD27 and IgD expression within the B cells define naïve (CD27^-^IgD^+^), switched memory (SM; CD27^+^IgD^-^), and double negative (DN; CD27^-^IgD^-^) B cells. Within these B cell subpopulations and total B cells the frequencies of D614G-RBD and Omicron-RBD reactive cells were estimated. The frequency of D614G RBD specific naïve B cells is very low for detection. Data was acquired with Cytoflex-30 cytometer and analyzed by FlowJo v10.3.

**Supplementary Figure S2.** Intensity levels of the indicated proteins from the proteomics data. Paired analyses of the intensities of the peptides corresponding to proteins differentially represented in the Post- and Pre- pools within the exercise (Top) and no-exercise (bottom) groups. Statistical comparisons were carried out with Mann-Whitney’s test or the Wilcoxon test.

**Supplementary Figure S3.** Plasma levels of CST3 and S100A8 analyzed by ELISA. (A) CST3 levels in PCC subjects before (Pre-E, Pre-NE) and at the end (Post-E, Post-NE) of the study period compared to no-PCC controls. (B) Paired analysis of pre- and post- CST3 levels within exercise and no-exercise PCC groups. (C) S100A8 levels in PCC subjects before (Pre-E, Pre-NE) and at the end (Post-E, Post-NE) of the study period compared to no-PCC controls. (D) Paired analysis of pre- and post- S100A8 levels within exercise and no-exercise PCC groups. Statistical comparisons were carried out with Mann-Whitney’s test or the Wilcoxon test.
